# Supplementary material for: Impact of implementation of front-of-package nutrition labeling on sugary beverage consumption and consequently on the prevalence of excess body weight and obesity and related direct costs in Brazil: An estimate through a modeling study
Source: PLoS One. 2023 Aug 11;18(8):e0289340. doi: 10.1371/journal.pone.0289340 (PMC10420370; doi:10.1371/journal.pone.0289340)
Supplement: S1 File — (DOCX) [file pone.0289340.s001.docx]

**Supporting Information for “Impact of implementation of front-of-package nutrition labeling on sugary beverage consumption and consequently on the prevalence of excess body weight and obesity and related direct costs in Brazil: An estimate through a modeling study”**

Natália Cristina de Faria^1^, Gabriel Machado de Paula Andrade^2^, Cristina Mariano Ruas^3^, Rafael Moreira Claro^4,5^, Luíza Vargas Mascarenhas Braga^1^, Eduardo Augusto Fernandes Nilson^5,6^, Lucilene Rezende Anastácio^1^*

1 - Post-Graduate Program in Food Science, Faculty of Pharmacy, Universidade Federal de Minas Gerais (UFMG), Belo Horizonte, Brazil. https://orcid.org/0000-0001-6716-9597; https://orcid.org/0000-0002-6822-7688; http://orcid.org/0000-0002-2269-0722

2 - Chemical Engineering Department, Universidade Federal do Rio de Janeiro (UFRJ), Rio de Janeiro, CEP 21941-972, Brazil. https://orcid.org/0000-0003-2059-278X

3 - Department of Social Pharmacy, Faculty of Pharmacy, Universidade Federal de Minas Gerais (UFMG), Belo Horizonte, Brazil. https://orcid.org/0000-0003-0275-8416

4 - Department of Nutrition, School of Nursing, Universidade Federal de Minas Gerais (UFMG), Belo Horizonte, Brazil. http://orcid.org/0000-0001-9690-575X

5 - Centre for Epidemiological Research in Nutrition and Health (NUPENS), University of Sao Paulo, Sao Paulo 05508-060, Brazil

6 - Oswaldo Cruz Foundation (Fiocruz) Brasília, Brasilia 70904-130, Brazil. https://orcid.org/0000-0002-2650-4878

*Corresponding author: Lucilene Rezende Anastácio

E-mail: lucilene.rezende@gmail.com ; https://orcid.org/0000-0002-2269-0722

**1. Database**

The VIGITEL Brazil 2019 (surveillance of risk and protective factors for chronic diseases by telephone survey) [1] was used in this study in order to model the different scenarios. Although there are other populational-based studies performed regularly in Brazil [2,3], none of them is are annual as VIGITEL. Other studies assess food consumption using the 24-hour recall, which is a method that requires several days of evaluation to estimate the habitual consumption of the participant, and depends on the interviewer [4]. Therefore, the 24-recall could underestimate the consumption of sugary beverages if performed in only one day. On the other hand, the VIGITEL focuses on the habitual consumption, which favored the choice of this database. Moreover, we wanted to include the temporal trends of reduction in the consumption of sugary beverages and the increase in the prevalence of obesity and overweight in the modeling estimates; thus, only a database such as VIGITEL could be used.

The VIGITEL survey, whose sample includes participants who live in all the Brazilian state capitals and in the Federal District, has been performed yearly since 2006 by telephone calls. It consists of a questionnaire that investigates various characteristics including food consumption, anthropometric (weight and height), and sociodemographic (age group, gender, age) ones, which were variables of interest in this study. The variables we used from the database are identified and described in S1 Table. The VIGITEL database is of public access, does not contain the identification of the participants and is available at: <http://svs.aids.gov.br/download/Vigitel/>.

S1 Table. Variables acquired from the VIGITEL database and used in the study.

| Variable | Variable description | Code |
| --- | --- | --- |
| Replica | Primary unit of analysis | NA |
| Year | Year VIGITEL was performed | NA |
| Q6 | Age (years) | NA |
| Q7 | Gender | 1 - male  2 - female |
| Q9 | Weight (kg) | 777 – does not know*  888 - does not know * |
| Q11 | Height (cm) | 777 - does not know *  888 - does not know * |
| Q14 | Pregnant | 1 - yes*  2 - no  777 - does not know |
| Q29 | How many days a week do you usually drink soft drinks or artificially flavored juice drinks? | 1 - 1 to 2 days a week  2 - 3 to 4 days a week  3 - 5 to 6 days a week  4 – Every day (including Saturday and Sunday)  5 – almost never*  6 - never* |
| Q30 | What type? | 1 - regular  2 - diet/ light/ zero*  3 - both |
| Q31 | How many glasses/cans do you usually drink per day? | 1 - glass/can per day  2 - glasses/cans per day  3 - glasses/cans per day  4 - glasses/cans per day  5 - glasses/cans per day  6 - 6 or more glasses/cans per day  7 – does not know* |
| Weight (through raking) | Expansion factor | NA |

NA: not applicable. *Participants who answered this code were excluded from this study.

**2. Estimate of the consumption of sugary beverages**

First, the database was adjusted according to the inclusion criteria of the study using the variables Q6, Q7, Q9, Q11, Q14, Q29, Q30, Q31. After that, the consumption of sugary beverages was estimated. The beverages included in this study were soft drinks or artificial juices [1]. In the VIGITEL study, there was analysis of the validation and reproducibility of the evaluation of the daily or almost daily consumption of soft drinks in random sub samples (n=112 and n=109) from the total of participants (N=2,204) for adults of 18 years of age or older in 2005. Evaluation of daily, or almost daily, consumption of soft drinks was among the indicators assessed. In order to analyze reproducibility, the results obtained in the original telephone interview were compared to the results obtained in another telephone interview performed between 7 and 15 days after the original one. The Kappa coefficients showed the results completely agree with each other (all: 0.77; men: 0.83; women: 0.72). In order to analyze validity, the results obtained from the telephone interview were compared to three 24-hour recalls performed 15 days after the original interview. Reasonable specificity values (all: 94.1%; men: 93.3%; women: 94.6%) and sensitivity values (all: 87.5%; men: 50%; women: 100%) were observed for the consumption of soft drinks [5].

Three questions from the VIGITEL questionnaire were used to estimate the consumption of sugary beverages. The question identified as Q29 in the VIGITEL database was used: “How many days a week do you usually drink soft drinks or artificial juices?” (1 to 2 days a week; 3 to 4 days a week; 5 to 6 days a week; every day, including Saturday and Sunday; almost never; never). Participants who answered “almost never” and “never” were excluded from this study. The question identified as Q30, which investigates the type of beverage consumed (regular, diet/light/zero, both), was also used. Participants who answered “diet/light/zero” were excluded. The question about the type of beverage consumed was omitted in the years of 2012, 2013 and 2014. The question identified as Q31 was used: “How many glasses/cans do you usually drink per day?” (1, 2, 3, 4, 5, 6 or more, does not know). Participants who did not know the quantity consumed were excluded.

To estimate the quantity of sugary beverages consumed per day, it was necessary to calculate the average frequency of consumption (AF) reported by the participants. Thus, the average of each interval of frequency of soft drink consumption reported by the participants was calculated (for one to two days a week, the value 1.5 was used; for three to four days a week, the value 3.5 was used; for five to six days a week the value 5.5 was used; and for every day, the value 7 was used). Furthermore, the average between a can (350mL) and a glass (150mL) of soft drink was calculated, which resulted in 250mL. Later, the estimated quantity of sugary beverages consumed per day (mL) by each individual included in the study was estimated through the frequency (question Q29 and AF), and quantity (question Q31) multiplied by 250 mL, and divided by the number of days in a week (7), as follows:

*Estimated quantity of sugary beverages consumed per day (mL) = Q29 x AF x Q31 x 250* (1)

*7*

The quantity of sugary beverages consumed, in mL, was converted into grams (g) by using the density value of 1.04 [6]. The Brazilian Food Composition Table (*Tabela Brasileira de Composição de Alimentos-TBCA*) [7] was used to inform the energy and sodium contents of the soft drink. The item “SOFT DRINK, REGULAR, WITH SUGAR (AVERAGE OF DIFFERENT SAMPLES),” which contained 38kcal and 8.12mg of sodium in 100g of soft drink (TBCA, Universidade de São Paulo - USP, Food Research Center – FoRC, Version 7.1. São Paulo, 2020, Accessed on: April 20, 2021, available at: http://www.fcf.usp.br/tbca). This way, the ingestion of calories and sodium from drinks was estimated.

**3. Public health policies that have impacted/will impact the consumption of sugary beverages in Brazil and Chile**

3.1 Brazilian legislation on front-of-package nutrition labeling

In Brazil, after much discussion, the legislation on front-of-package nutrition labeling was updated and implemented in a mandatory way. Such legislation was approved in October, 2020 by the Resolution of the Collegiate Board of Directors - RDC Nº 429 [8], which deals with the nutrition labeling of packaged foods, and Normative Instruction Nº 75 [9], which establishes technical requirements for declaration of nutritional labeling of packaged foods. Brazil has adopted the black magnifying glass design (S1 Fig) with the statement “high in” placed on the top half of the front panel of the food product when it presents excess added sugars, saturated fat and/or sodium. The limits for the nutrient profile can be seen in S2 Table. The legislation establishes that FoPNL must be used with one magnifying glass with the statement “high in” for each nutrient that surpasses the limits established for each eligible food.

S1 Fig – Front-of-package labeling with black magnifying glass design adopted by the Brazilian legislation, according to the Normative Instruction Nº 75 of 2020. Statements “high in” from left to right: added sugar; sodium; saturated fat; added sugar, saturated fat and sodium in the same product.

Source: Normative Instruction Nº 75, Brazil (2020) [9].

S2 Table – Profile of nutrients and their limits for liquid foods adopted by the Brazilian legislation.

| Nutrient per 100mL of liquids | Equal or higher quantity |
| --- | --- |
| Sodium (mg) | 100 |
| Added sugars (g) | 7.5 |
| Saturated fats (g) | 3 |

Source: Normative Instruction Nº75, Brazil (2020) [9].

3.2 Public policies that impacted sugary beverages in Chile

Chile has implemented, in a mandatory way, a set of public policies that complement each other with the intention to avoid the increase in noncommunicable chronic diseases related to diets. Such policies consisted of FoPNL in a warning format (black octagon design – S2 Fig), restrictions on marketing targeting children, as well as the prohibition of sales of packaged foods/drinks that present high contents of calories/free sugars/sodium/saturated fat. The implementation of the legislation started in 2016 and developed progressively in three phases. The profile of nutrients and their respective limits used for liquid foods are shown in S3 Table.

S2 Fig – Front-of-package nutrition labeling in a warning model and black octagon design adopted by Chilean legislation number 20,606. From left to right: high in sugars, high in saturated fats, high in sodium, high in calories.

Source: Adapted from Corvalan et al., 2018 [10].

S3 Table – Profile of nutrients and their limits for liquid foods adopted by the Chilean legislation according to each implementation phase.

| Nutrient per 100mL of liquids | June 26 of 2016 | June 26 of 2018 | June 26 of 2019 |
| --- | --- | --- | --- |
| Energy (kcal) | 100 | 80 | 70 |
| Sodium (mg) | 100 | 100 | 100 |
| Total sugars (g) | 6 | 5 | 5 |
| Saturated fats (g) | 3 | 3 | 3 |

Source: Law 20,606, Chile (2012) [11].

**4. Parameters of the change in energy and sodium in the modeled scenarios**

4.1 Base scenario – Estimation of the tendency of sugary beverages consumption

The temporal trend, concerning the consumption of sugary beverages, of 12 years from the VIGITEL survey was considered (between the years of 2007 and 2019, except for the year 2017, available at: http://svs.aids.gov.br/download/Vigitel/. The 2017 database was excluded, once the question used to analyze the consumption of sugary beverages was different from the one used in the other years. In 2017, the question was about the quantity of sugary beverages consumed on the day prior to the survey, while in the other years it was about the regular quantity consumed in one day.

With all the data adjusted according to the inclusion criteria of the study, the analysis of the proportion of Brazilians who consume sugary beverages and the estimated quantity/day of energy and sodium was performed for the years aforementioned. The sample weights from the VITIGEL survey were used in all editions and for each parameter analyzed in this study. Afterwards, linear regression was performed to evaluate the temporal trend of the number of sugary beverage consumers and the quantity/day of sodium and energy consumed. Such regression was also used to project the numbers up to 2024. The results were used in the base scenario of the modeling, both in isolation and associated with the other modeled scenarios. S3 Fig shows the average proportion of Brazilians who live in the Brazilian state capitals and consume sugary beverages and its projection up to 2024. S4-S5 Figs show the average consumption of energy and sodium from sugary beverages between the years of 2007 and 2019 (except 2017) and the projections up to 2024, respectively, among Brazilian consumers of such beverages.

S3 Fig – Proportion of Brazilians who lived in the Brazilian state capitals and consumed sugary beverages between the years of 2007 and 2019 (expect 2017) and its projection up to 2024.

S4 Fig – Average consumption of energy from sugary beverages by Brazilians who lived in the Brazilian state capitals and consumed such beverages between the years of 2007 to 2019 (except 2017) and its projection up to 2024.

S5 Fig – Average consumption of sodium from sugary beverages by Brazilians who lived in the Brazilian state capitals and consumed such beverages between the years of 2007 to 2019 (except 2017) and its projection up to 2024.

4.2 Scenario 1 – Estimation of the effect of front-of-package nutrition labeling on the purchase of sugary beverages

Scenario 1 consisted of the association of the base scenario with the results from the study by Taillie et al. (2021), which was a longitudinal study that analyzed the purchase of food and beverages before and after the implementation of the first phase of the Chilean legislation (from January 2015 to December 2017) [12]. Longitudinal data on purchases made by 2,381 households with individuals of all ages were analyzed. Once the analysis was performed regarding households, the results presented were not stratified by age. The beverages included in the study by Taillie et al. [12] are described in S4 Table. By comparing the different periods of implementation, a significant decrease in purchases regarding energy, sugar, saturated fats and sodium could be seen. S5 Table shows that there was a reduction in the purchase of drinks labeled “high in,” with some compensation, compared to the purchase of products without FoPNL. In this study, we considered that the decrease in the purchase of beverages converted into decrease in consumption. Therefore, the results concerning the purchase of beverages considering the compensation observed were used in the modeling scenario. We chose to use the results found by Taillie et al. [12] because: 1) they evaluated a policy that has already been implemented in Chile and it included FoPNL; 2) some compensation regarding the purchase of beverages was considered in the results. However, in that study, there was no possibility to obtain data about people in the same age group as the ones in the VIGITEL survey.

S4 Table – Beverages included in the study by Taillie et al. (2021).

| Sub-category | Description and examples |
| --- | --- |
| Soda | Carbonated soft drinks |
| Industrialized fruit and vegetable drinks | Industrialized fruit-flavored drinks, including powdered drinks, nectars, and ready-to-drink ones |
| Dairy-based beverages and dairy substitutes | Plain and flavored milks, dairy substitutes, and other dairy drinks, including powdered and ready-to-drink ones |
| Waters | Plain and flavored waters, mineral water, sparkling water, including powdered and ready-to-drink ones |
| Coffee and tea | Instant coffee, roasted coffee, ground coffee, RTD tea, and powdered tea |
| 100% fruit and vegetable juice | 100% fruit and vegetables juices, no additives |
| Sports drinks | Powdered and ready-to-drink sports drinks |
| Formula | Toddler/child formula, infant/baby formula, and maternal/elderly formula |

S5 Table – Average differences in the purchase of beverages, in energy and sodium, before and after implementation of the policies observed by Taillie et al. (2021).

| Evaluated parameter | Beverages “high in” | Beverages without FoPNL | General beverage purchase |
| --- | --- | --- | --- |
| Energy - kcal/per capita/day (95%CI) | -16.3  (–18.7 to –13.9) | +6.3  (4.1 to 8.5) | -10.0  (–13.4; –6.6) |
| Energy (%) | -31.3 | +12.6 | -9.9* |
| Sodium - mg/per capita/day (95%CI) | -7.0  (–8.0 to –6.3) | +3.7  (1.6 to 5.9) | -3.4  (–5.8 to –1·1) |
| Sodium (%) | -43.3 | +7.6 | -5.2* |

*values used in modeling scenario 1 associated with the base scenario.

4.3 Scenario 2 – Estimation of the effect of front-of-package nutrition labeling on the purchase of sugary beverages associated with reformulation

Another expected consequence of the implementation of FoPNL is the reformulation of foods and beverages performed by the food industry, as described in other studies [13–15] and which already seems to be happening in Brazil [16]. Thus, for scenario 2, scenario 1 was associated with the results found in the study by Kanter et al. (2019), who evaluated beverage reformulation during the pre-implementation period of the Chilean legislation (February 2015 – February 2016). The beverages included in the study by Kanter et al. were: vegetable and fruit juices, nectar, flavored beverages, flavored waters, sorbet, sports drinks, soft drinks, flavored powder drinks and others [14]. It was observed that the composition of beverages changed; however, while the energy content decreased, the sodium content increased. S6 Table shows the change observed. We chose to use the results found by Kanter et al. (2019) once they evaluated the pre-implementation period in Chile, which is a period similar to the one Brazil is going through. In Brazil, the implementation of FoPNL started in October 2022, and the deadline for most products to be adequate until October 2023 [8,9].

S6 Table – Nutritional composition of beverages (median, interquartile range and average percentage changes) regarding the contents of energy and sodium during the pre-implementation period of the Chilean legislation observed by Kanter et al., (2019).

| Evaluated parameter | 2015 | 2016 | Average percentage changes |
| --- | --- | --- | --- |
| Energy - kcal/100mL | 30  (12, 44) | 28  (11, 44) | -1.6* |
| Sodium - mg/100mL | 10  (6, 17) | 10  (5, 17) | +1.8* |

*values used in modeling scenario 2 associated with scenario 1.

4.4 Scenarios of the sensitivity analysis

Alternative scenarios were used for the sensitivity analysis; one based on an experimental market study that analyzed the effect of FoPNL on the purchase of beverages (scenario 3), and another associated with a possible reformulation of products (scenario 4).

The experimental study used in scenario 3 was performed by Acton et al. (2019), who evaluated the effect of FoPNL on the purchase of beverages, in energy and sodium. It consisted of an experimental market study performed in three Canadian cities in which the participants purchased a beverage. Twenty options of beverages, sweetened with sugar or sweetener, were presented to the participants, such as soft drinks, sports drinks, flavored waters, ready-to-drink tea, fruit juice, milk, chocolate milk and water. They tested a control group (without labeling) and a group using a red warning circle label with the statement “high in” for beverages that exceeded the limits of sugar, sodium or saturated fats (S6 Fig). The beverages that presented quantities equal to or higher than 15g/serving of free sugars and/or 345mg/serving of sodium and/or 3g/serving of saturated fat received FoPNL, according to the criterion established by Health Canada [17].

S6 Fig – Front-of-package nutrition labeling in red warning circle design model used in the experimental market study by Acton et al. (2019).

Source: Adapted from Acton et al. (2019) [18].

The results used in this study came from a sub-sample of the study by Acton et al., which consisted only of adults aged over 18 years (n=1,213) [18,19]. S7 Table shows the variations in purchase between the intervention and control groups, in energy and sodium. We chose to use this study for the sensitivity analysis because the participants were in the same age group as the individuals included in the VIGITEL database. For scenario 4, the results from scenario 3 were associated with those observed by Kanter et al. (2019), who evaluated the reformulation of beverages in Chile (described in item 4.3).

S7 Table – Purchase of beverages observed in the experimental market study by Acton et al., (2019), in calorie and sodium average and percentage average variation, between the intervention and control groups.

| Beverage composition | Without labeling | “High in” labeling | Average variation (percentage) |
| --- | --- | --- | --- |
| Energy - kcal | 102.0 | 91.3 | -10.5* |
| Sodium – mg | 69.2 | 65.4 | -5.5* |

*values used in modeling scenario 3 for sensitivity analysis associated with base scenario.

**5. Estimation of the effect of changes in the intake of energy and sodium on weight and body mass index in Brazilians who consume sugary beverages**

5.1 Estimation of the change in sugary beverage intake (in energy and sodium) after the implementation of front-of-package nutrition labeling by Brazilians

The change in energy and sodium intake for each individual (k), in relation to the intake of sugary beverages, was calculated for the different FoPNL implementation scenarios and for the base scenario (the term reduction was used in the following equations). Therefore, energy variation (*ΔEI*) and sodium variation (*ΔNa*) were calculated as follows:

*ΔEI_k_ = Intake of beverages in energy_k_ x reduction* (2)

*ΔNa_k_ = Intake of beverages in sodium_k_ x reduction* (3)

5.2 Estimation of the change in body weight of Brazilians due to decreased intake of sugary beverages attributed to the implementation of front-of-package nutrition labeling

The Dynamics of Human Body Weight Change model, proposed by Hall et al. (2011), was used in adults to estimate the possible effect of the changes in energy and sodium intake on body weight. Thus, the bw package was used in R which is coded for the model [20]. In order to estimate the change in body weight (bw), the differences in energy and sodium intake were considered in addition to gender, age, initial body weight (bw_k_), height of each individual and time in number of days (t), as shown in the following equation:

*BW_k_(t) = BW _k_^model^ = (t + age_k_; gender_k_; height_k_; bw_k_(_initial_); ΔEI_k_; ΔENa_k_) (4)*

During the simulated period, the changes in energy and sodium intake that may be promoted by FoPNL were considered constant for the different scenarios in a temporal horizon of 5 years. Other interventions may cause changes in energy and sodium intake, which may alter the effects modeled for the different scenarios, such as new policies, taxes, other interventions in health, among others [21,22].

5.3 Estimation of the change in the body mass index of Brazilians who consume sugary beverages attributed to a decrease in the intake of sugary beverages promoted by the implementation of front-of-package nutrition labeling

A new BMI was calculated for each individual using the change in body weight estimated in the previous step, as follows:

*BMI_k_ = BW_k_(initial) - BW_k_(t)* (5)

*Height^2^*

5.4 Estimation of the change in prevalence of obesity for the Brazilians who consume sugary beverages

The participants were categorized into individuals with excess body weight (BMI greater than or equal to 25kg/m^2^) and obesity (BMI greater than or equal to 30kg/m^2^), according to the cutoff point adopted by WHO [23], in all the modeled scenarios. Therefore, a new proportion of cases of excess weight and obesity was estimated for the 5 years modeled in all scenarios.

All information was projected considering the use of complex sample functions (survey), using the variables weight and primary unit of sampling incorporated in the analysis with the Epi Info software, version 7.2, as described in the Analysis Manual of VIGITEL [24].

**6. Estimation of the prevalence of excess weight and obesity in Brazilians who consume sugary beverages and impact on the general population of Brazil in the 20-59 age group**

6.1 Temporal trend of the prevalence of excess weight and obesity in Brazilians who consume sugary beverages

Although there is a tendency towards reduction in the consumption of sugary beverages [25], there is also an increase in the prevalence of body weight excess and obesity [26]. Therefore, for all the modeled scenarios, the temporal trend of prevalence of excess weight and obesity based on the VIGITEL survey (from 2007 to 2019, except 2017) was considered with the same inclusion criteria of the study, which were analyzed with linear regression and projection up to 2024. S7-S8 Figs show the average proportion (between 2007 and 2019, except 2017) and the projections, up to 2024, of Brazilians who live in the Brazilian state capitals, are sugary beverages consumers, and present body weight or obesity, respectively. S3 Fig, previously presented, showed the temporal trend of Brazilians who consume sugary beverages based on the VIGITEL survey (from 2007 to 2019, except 2017) obtained through linear regression and the projection up to 2024.

S7 Fig – Proportion of Brazilians who consume sugary beverages and have excess body weight living in Brazilian state capitals between the years of 2007 and 2019 (except 2017) and its projection up to the year 2024.

S8 Fig - Proportion of Brazilians who consume sugary beverages and are obese living in Brazilian state capitals between the years of 2007 and 2019 (except 2017) and its projection up to the year 2024.

6.2 Temporal trend of the prevalence of excess weight and obesity in consumers of sugary beverages extrapolated to the Brazilian population

Initially, a projection of the Brazilian population up to 2024 in the same age group of the study (20 to 59 years) was used. It was obtained from census information from the Brazilian Institute of Geography and Statistics, available at: https://www.ibge.gov.br/apps/populacao/projecao/in. Afterwards, the number of Brazilians who consume sugary beverages was estimated based on the temporal trend estimated in the previous editions of VIGITEL (S8 Table). Moreover, the number of adult Brazilians who consume sugary beverages and have excess body weight and obesity was estimated up to 2024 based on the temporal trends described in S8 Table.

*No of Brazilian consumers of sugary beverages_(year)_ = Adult population_(year)_ x Temporal trend%1_(year)_* (6)

*No of Brazilians with excess body weight _(year)_ = No of Brazilian consumers of sugary beverages_(year)_ x Temporal trend %2_(year)_* (7)

*No of obese Brazilians _(year)_= No of Brazilian consumers of sugary beverages_(year)_ x Temporal trend %3_(year)_* (8)

S8 Table – Estimations of Brazilian who consume sugary beverages and the prevalence of excess body weight and obesity up to 2024 based on the temporal trends and projections according to the VIGITEL survey.

| Temporal trends: | 2020  %  n | 2021  %  n | 2022  %  n | 2023  %  n | 2024  %  n |
| --- | --- | --- | --- | --- | --- |
| Sugary beverage consumers (1) | 45.3  54,043,044 | 44.5  53,447,776 | 43.7  52,747,104 | 42.9  52,004,688 | 42.1  52,203,903 |
| Excess body weight (2) | 60.1  32,469,061 | 61.4  32,816,934 | 62.7  33,082,983 | 64.0  33,303,802 | 65.4  34,120,471 |
| Obesity (3) | 22.8  12,294,793 | 23.6  12,597,641 | 24.4  12,859,744 | 25.2  13,099,981 | 26.0  13,573,015 |

6.3 Estimation of the reduction of cases of excess body weight and obesity extrapolated for the Brazilians who consume sugary beverages.

The proportions of excess weight and obesity along the five years and in all the scenarios were extrapolated to the Brazilians who consume sugary beverages in order to estimate the new prevalence.

*No of cases of excess weight _(year)_ = No of Brazilian consumers of sugary beverages _(year)_ x proportion of excess weight in each modeled scenario _(year)_* (9)

*No of cases of obesity _(year)_ = No of Brazilian consumers of sugary beverages _(year)_ x proportion of excess weight in each modeled scenario _(year)_* (10)

The difference concerning the number of cases of excess weight and obesity between estimated scenarios 1, 2, 3 or 4 and the base scenario was calculated to estimate the number of reduced cases of excess weight and obesity attributed to FoPNL according to each scenario.

*Δ cases of excess weight _(year)_ = No of cases of excess weight _(year)_ in each scenario (1, 2, 3 or 4) - No of cases of excess weight _(year)_ in the base scenario* (11)

*Δ number of reduced cases of obesity _(year)_ = number of cases of obesity _(year)_ in each scenario (1, 2, 3 or 4) – number of cases of obesity _(year)_ in the base scenario* (12)

6.4 Estimation of the impact of the reduction of excess body weight and obesity cases on the prevalence trend of excess weight and obesity in the Brazilian population

The difference between the number of Brazilians who consume sugary beverages and have excess weight and are obese (step 6.1) and the number of excess weight and obesity cases obtained in step 6.2 was calculated.

*Estimation of the number of Brazilian with excess weight _(year)_ = number of Brazilians with excess weight _(year)_ - Δ excess weight cases _(year)_* (13)

*Estimation of the number of obese Brazilians _(year)_ = number of obese Brazilians_(year)_ - Δ obesity cases _(year)_* (14)

A new prevalence of excess weight and obesity was estimated for the population who consume sugary beverages and the impact on the total Brazilian population was estimated year by year (S9 Table). The impact caused by the quantity of reduced obesity cases was calculated using the ratio between the number of reduced cases to the Brazilian population in the age group of interest. The estimated results of the impact on the total Brazilian population are presented as percentage in S10 Table.

S9 Table – Estimations of the prevalence of excess body weight and obesity in Brazilian consumers of sugary beverages after the implementation of front-of-package labeling up to 2024, and sensitivity analysis.

| Estimated prevalence | 2020  % | 2021  % | 2022  % | 2023  % | 2024  % |
| --- | --- | --- | --- | --- | --- |
| Scenario 1  Obesity  Excess weight | 22.3  59.6 | 22.8  60.4 | 23.9  61.8 | 24.5  63.2 | 25.3  64.4 |
| Scenario 2  Obesity  Excess weight | 22.2  59.5 | 22.7  60.4 | 23.6  61.7 | 24.4  63.0 | 25.2  64.2 |
| Scenario 3  Obesity  Excess weight | 22.2  59.5 | 22.8  60.4 | 23.7  61.7 | 24.4  63.0 | 25.2  64.3 |
| Scenario 4  Obesity  Excess weight | 22.2  59.5 | 22.7  60.3 | 23.4  61.6 | 24.4  62.9 | 25.1  62.2 |

S10 Table – Estimations of the reduction in prevalence of excess body weight and obesity in the Brazilian population attributed to the implementation of the front-of-package up to 2024, and sensitivity analysis.

| Reduction in prevalence | 2020  pp | 2021  pp | 2022  pp | 2023  pp | 2024  pp |
| --- | --- | --- | --- | --- | --- |
| Scenario 1  Obesity  Excess weight | -0.19  -0.22 | -0.35  -0.42 | -0.21  -0.41 | -0.29  -0.36 | -0.32  -0.42 |
| Scenario 2  Obesity  Excess weight | -0.25  -0.26 | -0.37  -0.45 | -0.32  -0.44 | -0.35  -0.45 | -0.35  -0.48 |
| Scenario 3  Obesity  Excess weight | -0.25  -0.25 | -0.35  -0.44 | -0.29  -0.43 | -0.34  -0.42 | -0.32  -0.46 |
| Scenario 4  Obesity  Excess weight | -0.25  -0.27 | -0.37  -0.49 | -0.34  -0.47 | -0.35  -0.47 | -0.37  -0.48 |

**7. Impact on direct costs of obesity**

In order to obtain the direct costs with obesity for the public health system, we used the information described in the study by Nilson et al., 2020, who estimated the costs related to obesity for the SUS (Sistema Único de Saúde – Unified Health System) in Brazil in 2018. Costs regarding hospitalization, outpatient procedures, as well as medication provided by SUS for the treatment of obesity were considered. The results described in the study by Nilson et al. refer to the general adult population; however, the authors provided the estimate of the costs for the age group of 20 to 59 years, the same age group used in this study. The costs per capita regarding obesity were estimated based on the costs for the age group of 20 to 59 years. The costs related to the year of 2018 are described in S11 Table.

S11 Table – Estimations of the direct costs regarding obesity according to what Nilson et al. described for the total Brazilian population and for the age group of 20 to 59 years.

| Costs | 2018  (20-59 years) | 2018  (per capita) | 2018  obesity cases assisted by SUS (n) |
| --- | --- | --- | --- |
| Direct costs  (reais - R$) | 725,568,380.76 | 43.38 | 16,724,223 |
| Direct costs  (American dollar*) | 187,243,453.10 | 11.20 |  |

*Exchange rate in 2018: R$ 3.875.

The inflation rate IPCA (Índice de Preços ao Consumidor Amplo - Extended National Consumer Price Index), which was 1,082128, was used to correct the values from 2018 and 2019. Afterwards, the discount rate of 5% for each estimated year was used as recommended in Brazil, for the period between 2020 and 2024 as follows:

*Cost of obesity per capita _(year + 1)_ = Cost of obesity per capita _year_ (1 + 0.05)* (15)

The number of reduced obesity cases was multiplied by the value per capita of obesity costs to estimate the reduction in direct costs in health due to the implementation of FoPNL in Brazil in the different scenarios. The results were presented in dollars (exchange rate in 2019: R$4.03) and in PPP dollars (purchasing power parities, R$2.28).

**8. References**

1. Brasil. Ministério da Saude. Vigitel Brasil 2019 - Vigilância de fatores de risco e proteção para doenças crônicas por inquérito telefônico: estimativas sobre frequência e distribuição sociodemográfica de fatores de risco e proteção para doenças crônicas nas capitais dos 26 estados. 2020. Avaible: https://bvsms.saude.gov.br/bvs/publicacoes/vigitel_brasil_2019_vigilancia_fatores_risco.pdf.

2. IBGE. Pesquisa Nacional de Saúde 2019: informações sobre domicílios, acesso e utilização dos serviços de saúde. Instituto Brasileiro de Geografia e Estatistica- IBGE. 2020. Available: http://biblioteca.ibge.gov.br/visualizacao/livros/liv91110.pdf

3. IBGE. Pesquisa de Orçamentos Familiares 2017-2018: Análise do Consumo Alimentar Pessoal no Brasil. Ibge. 2020. Available: http://scholar.google.com/scholar?hl=en&btnG=Search&q=intitle:Pesquisa+de+Or?amentos+Familiares#0

4. Morimoto JM, Marchioni DML, Cesar CLG, Fisberg RM. Within-person variance for adjusting nutrient distribution in epidemiological studies. Rev Saúde Pública. 2011;45: 1–4. doi:https://doi.org/10.1590/S0034-89102011000300022

5. Monteiro CA, Moura EC, Jaime PC, Claro RM. Validity of food and beverage intake data obtained by telephone survey. Rev Saúde Pública. 2008;42. doi:DOI: 10.1590/s0034-89102008000400002

6. Moreira BG, Olivieri BS, Gouvêa PMP, Borges HA, Aibe V, Valente LCG. Diferenciação de bebidas alcoólicas e não-alcoólicas no sistema de medição de vazão (SMV). Curitiba; 2007. Available: https://www.researchgate.net/profile/Paula_Gouvea3/publication/283088780_DIFERENCIACAO_DE_BEBIDAS_ALCOOLICAS_E_NAO-ALCOOLICAS_NO_SISTEMA_DE_MEDICAO_DE_VAZAO_SMV/links/562a471008ae04c2aeb1814c/DIFERENCIACAO-DE-BEBIDAS-ALCOOLICAS-E-NAO-ALCOOLICAS-NO-SISTEMA

7. Universidade de São Paulo, Food Research Center F. Tabela Brasileira de Composição de Alimentos (TBCA). Versão 7.0. São Paulo; 2020. Available: http://www.fcf.usp.br/tbca

8. Brasil. Ministério da Saude. Resolução da Diretoria Colegiada - RDC n^o^ 429, de 8 de outubro de 2020. Avaible: https://www.in.gov.br/en/web/dou/-/resolucao-de-diretoria-colegiada-rdc-n-429-de-8-de-outubro-de-2020-282070599.

9. Brasil. Ministério da Saude. Instrução Normativa n^o^75, de 8 de outubro de 2020. Avaible: https://www.in.gov.br/en/web/dou/-/instrucao-normativa-in-n-75-de-8-de-outubro-de-2020-282071143.

10. Corvalán C, Reyes M, Garmendia ML, Uauy R. Structural responses to the obesity and non-communicable diseases epidemic: Update on the Chilean law of food labelling and advertising. Obes Rev. 2018;20: 367–374. doi:10.1111/obr.12802

11. Ministerio de Salud Chile, Subsecretaria de Salud Pública. Ley número 20.606 sobre composición nutricional de los alimentos y su publicidad. Chile; 2012 p. 5. Available: https://www.bcn.cl/leychile/navegar?idNorma=1041570

12. Taillie LS, Bercholz M, Popkin B, Reyes M, Colchero MA, Corvalán C. Changes in food purchases after the Chilean policies on food labelling , marketing , and sales in schools : a before and after study. Lancet Planet Heal. 2021;5: 526–533. doi:10.1016/S2542-5196(21)00172-8

13. Scarpelli DQ, Fernandes ACP, Osiac LR, Quevedo TP. Changes in Nutrient Declaration after the Food Labeling and Advertising Law in Chile: A Longitudinal Approach. Nutrients. 2020;12: 1–13. doi.org/10.3390/nu12082371.

14. Kanter R, Reyes M, Vandevijvere S, Swinburn B, Corvalán C. Anticipatory effects of the implementation of the Chilean Law of Food Labeling and Advertising on food and beverage product reformulation. Obes Rev. 2019;20: 129–140. doi:10.1111/obr.12870

15. Mhurchu CN, Eyles H, Choi Y-H. Effects of a Voluntary Front-of-Pack Nutrition Labelling System on Packaged Food Reformulation: The Health Star Rating System in New Zealand. Nutrients. 2017;9: 1–16. doi:10.3390/nu9080918

16. Tomaz LA. Uso de edulcorantes na produção de alimentos e bebidas: frequência e utilização segundo a RDC n^o^18/2008, no cenário de pré-implementação da nova norma brasileira de rotulagem nutricional. Universidade Federal de Minas Gerais. 2022.

17. Health Canada. Toward front-of-package nutrition labels for Canadians: Consultation document. Ottawa. Canada; 2016 p. 22. Available: https://www.canada.ca/content/dam/canada/health-canada/migration/health-system-systeme-sante/consultations/labels-nutrition-etiquetage/alt/labels-nutrition-etiquetage-eng.pdf

18. Acton RB, Jones AC, Kirkpatrick SI, Roberto CA, Hammond D. Taxes and front-of-package labels improve the healthiness of beverage and snack purchases : a randomized experimental marketplace. Int J Behav Nutr Phys Act. 2019;16: 1–15. doi:https://doi.org/10.1186/s12966-019-0799-0 RESEARCH

19. Basto-abreu A, Torres-alvarez R, Reyes-s F, Gonz R, Canto-osorio F, Colchero MA, et al. S1 Appendix for ” Predicting obesity reduction after implementing warning labels in Mexico : a modeling study ”. PLoS Med. 2020;S1 Appendix. doi.org/10.1371/journal.pmed.1003221.s003

20. Camacho-García-Formentí D, Zepeda-Tello R. bw: Dynamic Body Weight Models for Children and Adults. 2018. Available: https://rdrr.io/cran/bw/

21. Basto-Abreu A, Torres-Alvarez R, Reyes-Sánchez F, González-Morales R, Canto-Osorio F, Colchero MA, et al. Predicting obesity reduction after implementing warning labels in Mexico: A modeling study. PLoS Med. 2020;17: 1–14. doi:10.1371/journal.pmed.1003221

22. Blakely T, Cleghorn C, Mizdrak A, Waterlander W, Nghiem N, Swinburn B, et al. The effect of food taxes and subsidies on population health and health costs : a modelling study. Lancet Public Heal. 2020;5: e404–e413. doi:10.1016/S2468-2667(20)30116-X

23. WHO. WHO Ecport Committee on Physical Status: the Use and Interpretation of Anthropometry Physical status; the use and interpretation of anthropometry: report of a WHO expert committee. 1995. p. 854.

24. Ministério da Saúde, Secretaria de Vigilância em Saúde, Departamento de Vigilância de Doenças e Agravos Não Transmissíveis. Manual de Análise do Vigitel no Epi Info (versão 7.2.2.6). Brasília-DF; 2018. p. 23.

25. Figueiredo N, Maia EG, Da Silva LES, Granado FS, Claro RM. Trends in sweetened beverages consumption among adults in the Brazilian capitals, 2007-2016. Public Health Nutr. 2018;21: 3307–3317. doi:10.1017/S1368980018002161

26. Estivaleti JM, Habinger JG, Lobos J, Azeredo CM, Claro R, Ferrari G, et al. Time trends and projected obesity epidemic in Brazilian adults between 2006 and 2030. Sci Rep. 2022;12: 1–8. doi:10.1038/s41598-022-16934-5
