# Supplementary figures and images for: Impact of implementation of front-of-package nutrition labeling on sugary beverage consumption and consequently on the prevalence of excess body weight and obesity and related direct costs in Brazil: An estimate through a modeling study
Source: PLoS One. 2023 Aug 11;18(8):e0289340. doi: 10.1371/journal.pone.0289340 (PMC10420370; doi:10.1371/journal.pone.0289340)

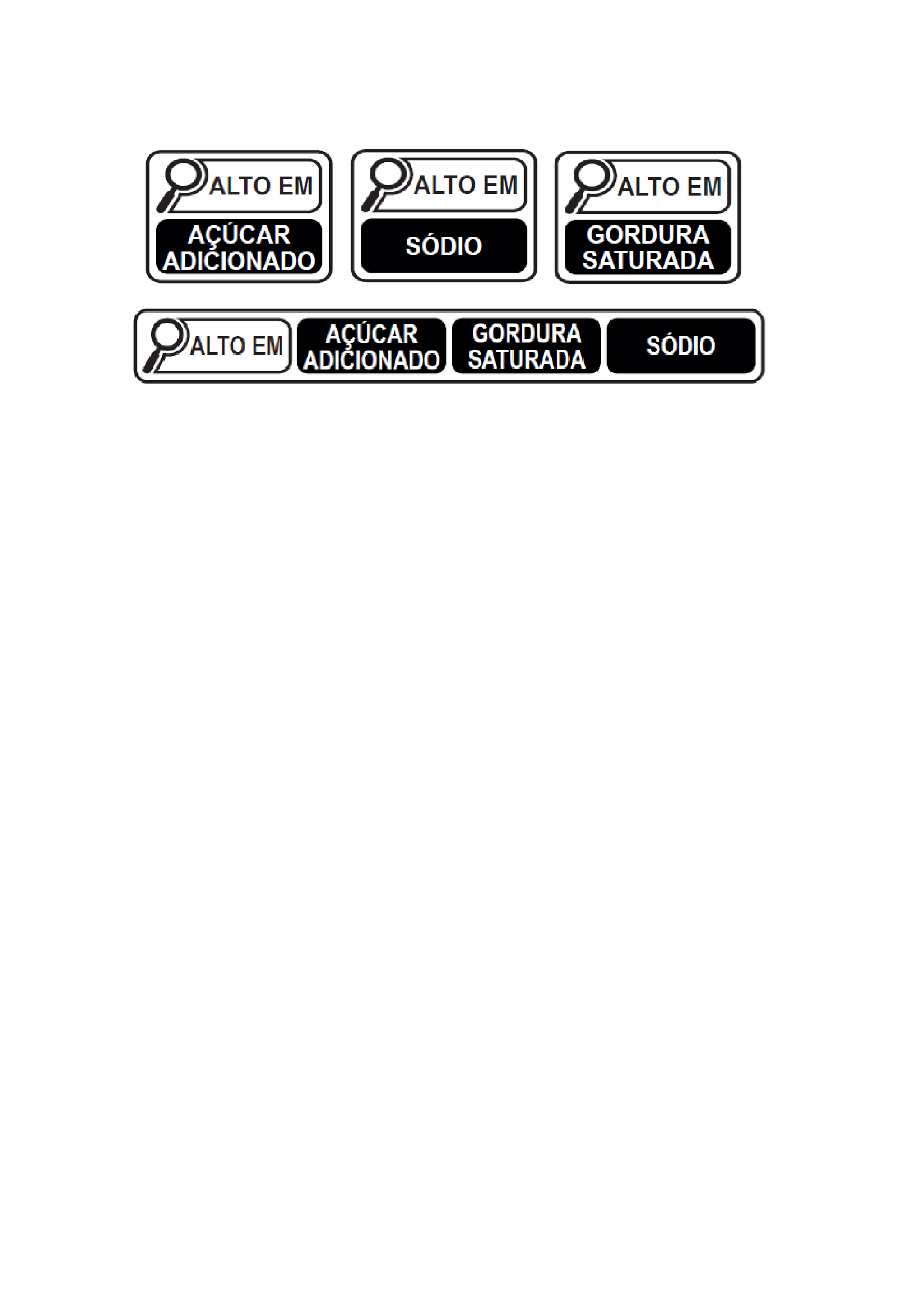

Supplement: S1 Fig — Statements “high in” from left to right: added sugar; sodium; saturated fat; added sugar, saturated fat and sodium in the same product. (TIF) [file pone.0289340.s002.tif]

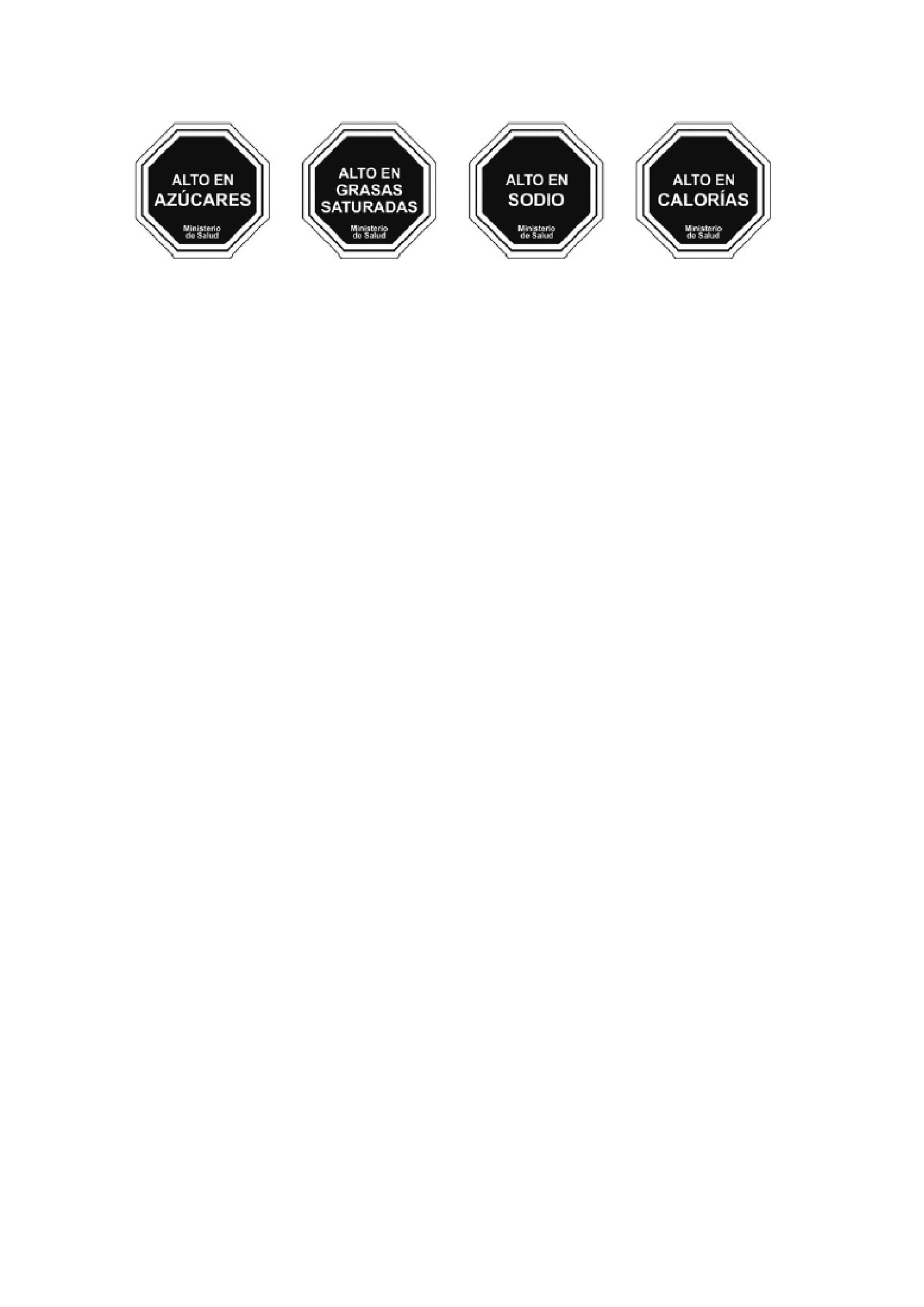

Supplement: S2 Fig — From left to right: high in sugars, high in saturated fats, high in sodium, high in calories. (TIF) [file pone.0289340.s003.tif]

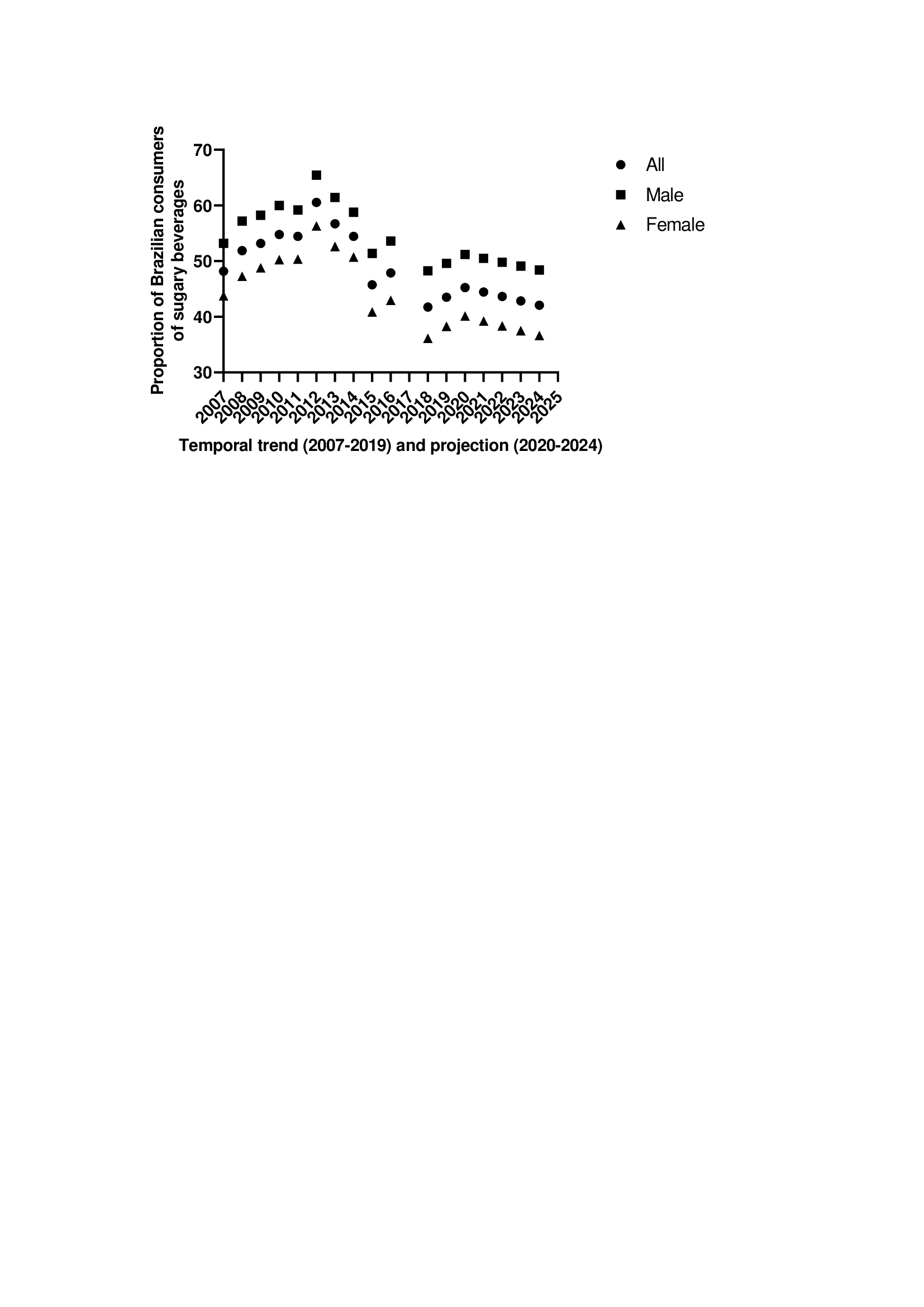

Supplement: S3 Fig — (TIF) [file pone.0289340.s004.tif]

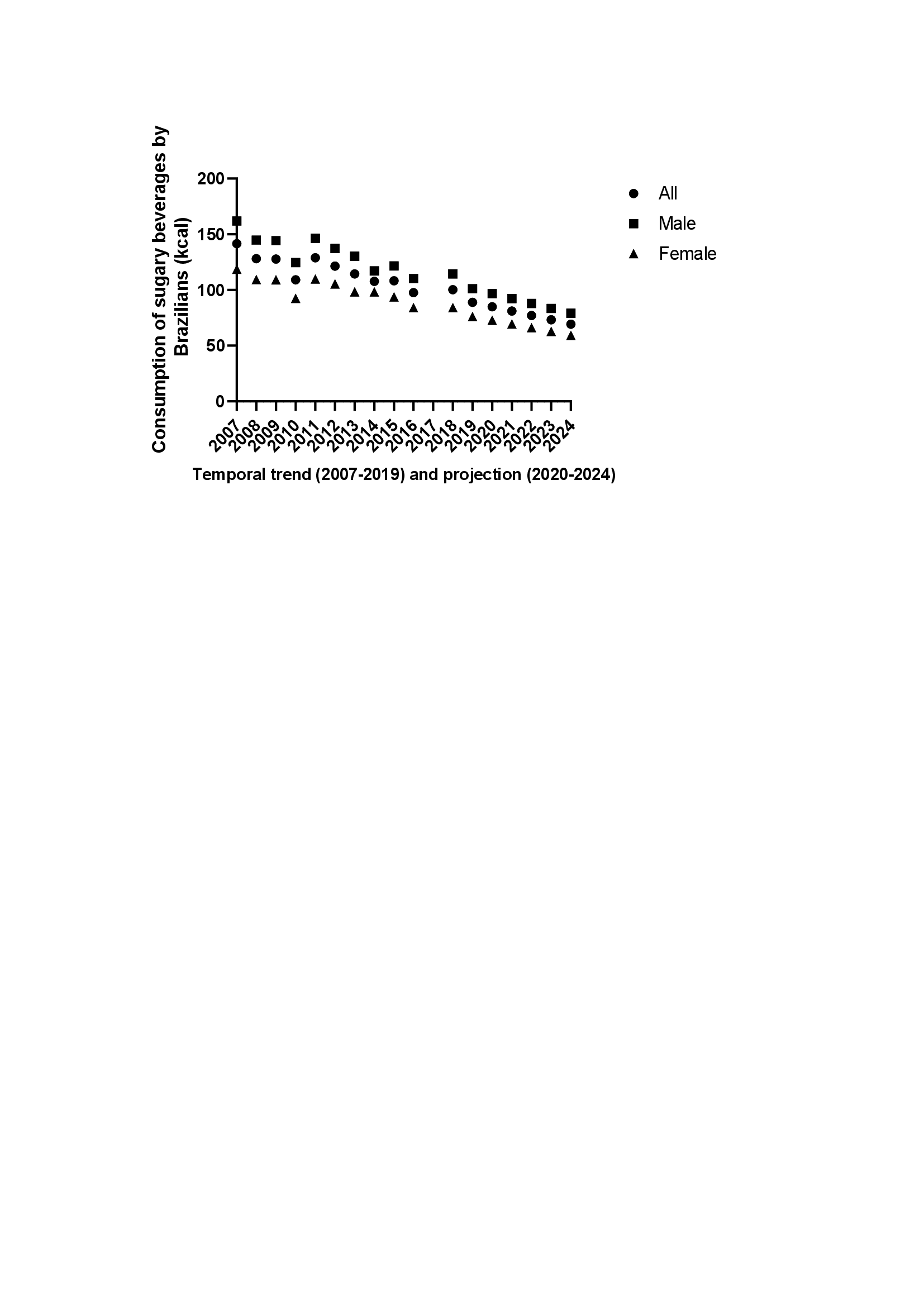

Supplement: S4 Fig — (TIF) [file pone.0289340.s005.tif]

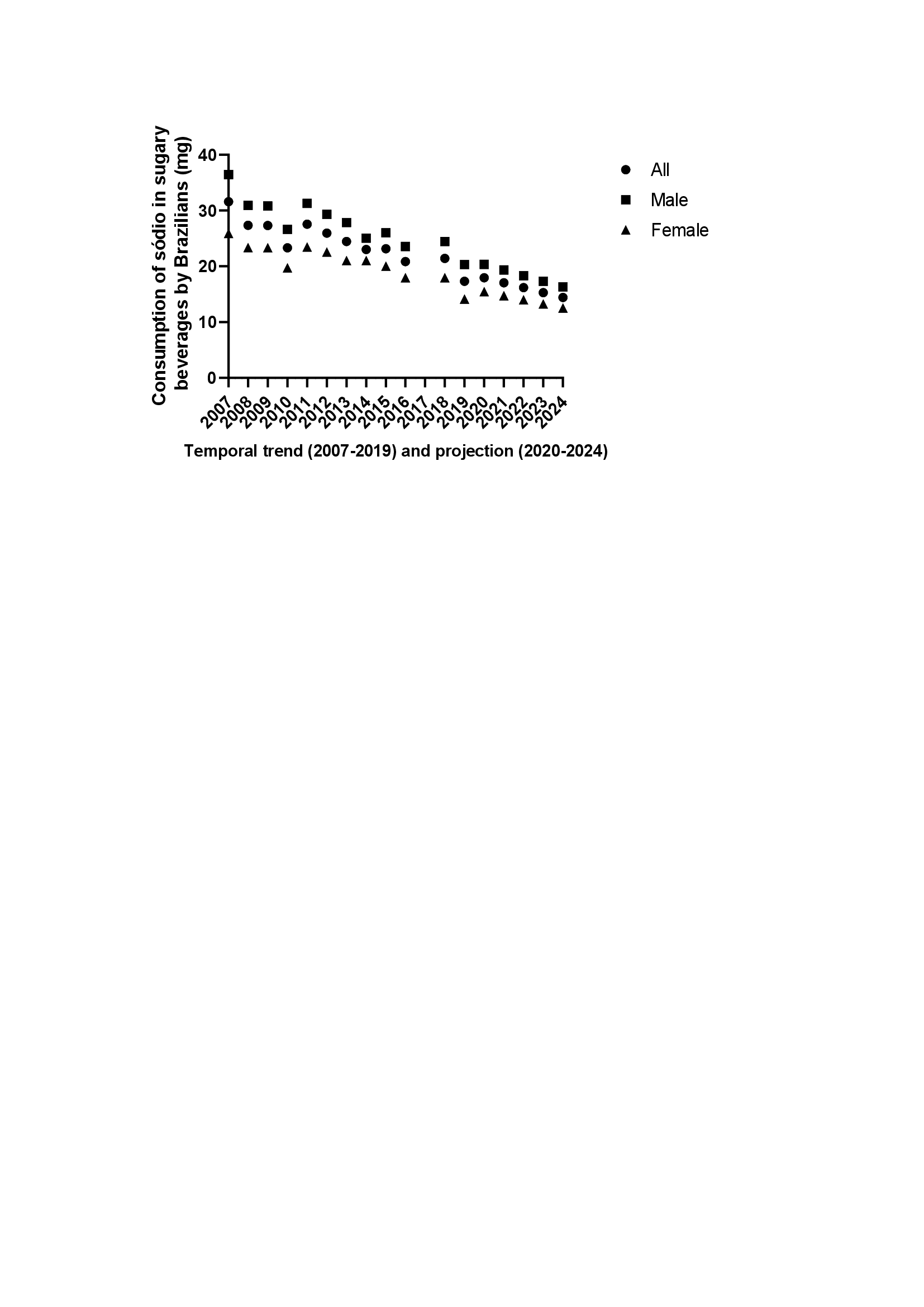

Supplement: S5 Fig — (TIF) [file pone.0289340.s006.tif]

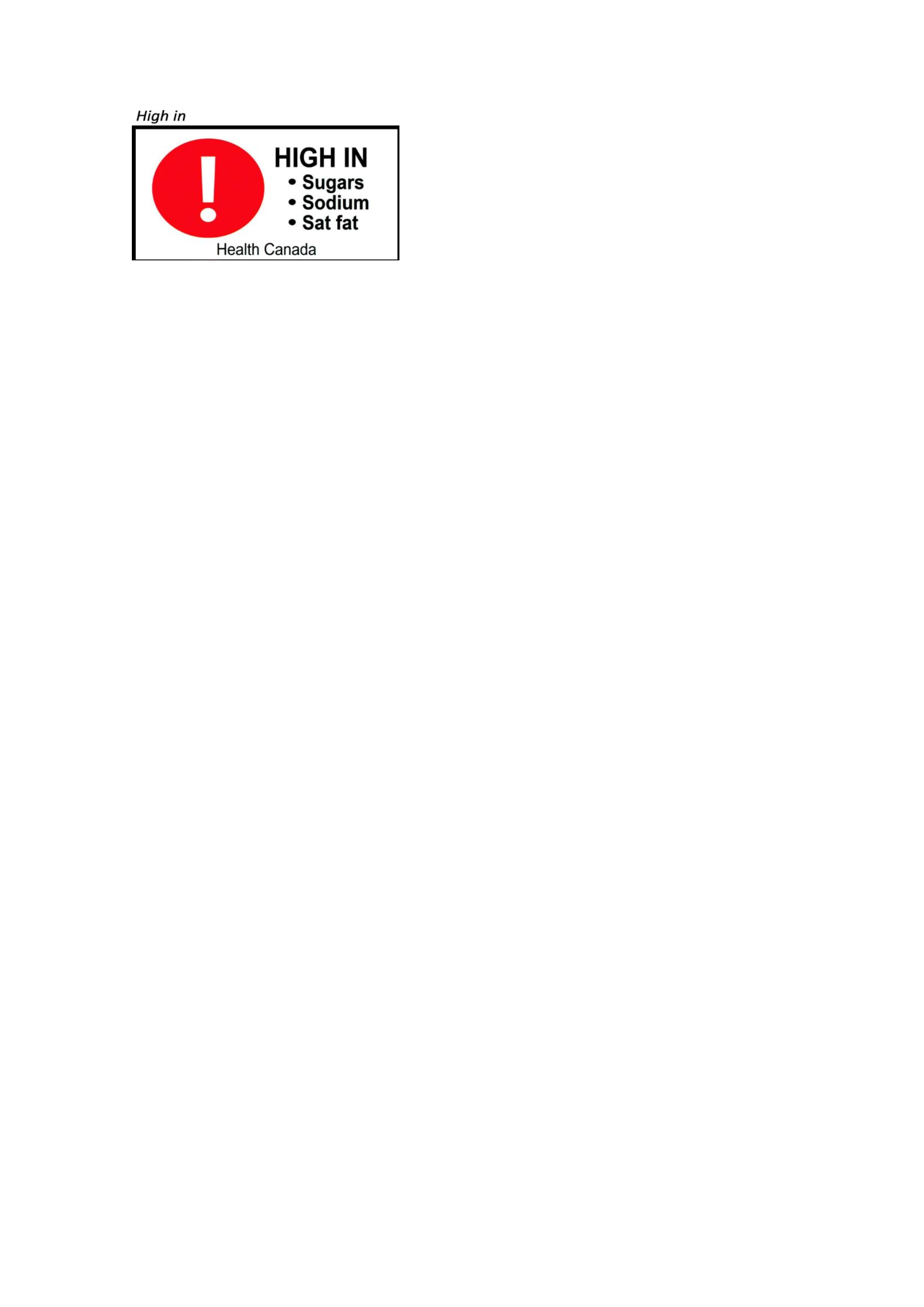

Supplement: S6 Fig — (TIF) [file pone.0289340.s007.tif]

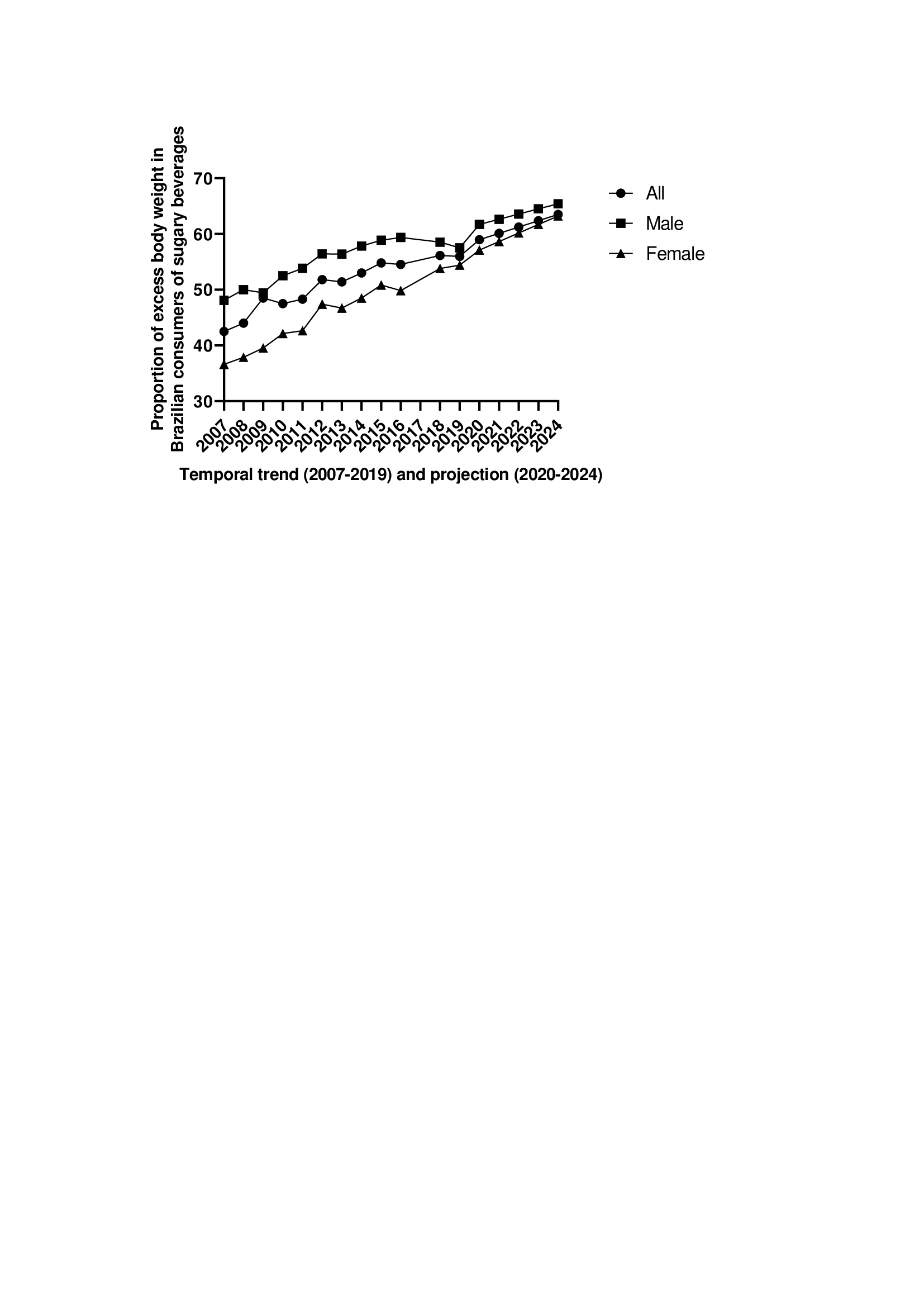

Supplement: S7 Fig — (TIF) [file pone.0289340.s008.tif]

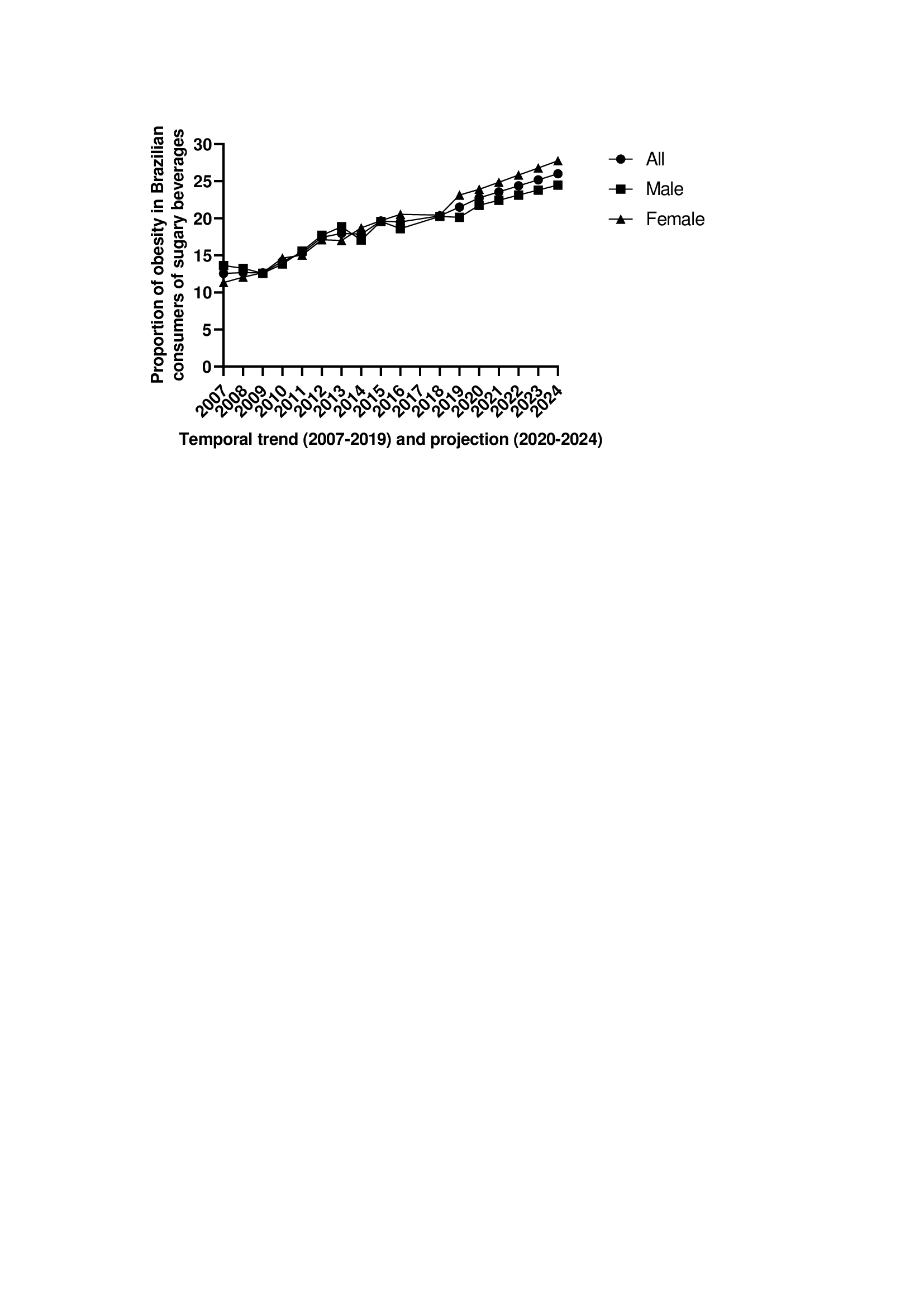

Supplement: S8 Fig — (TIF) [file pone.0289340.s009.tif]
