# Supplementary material for: Impact of implementation of front-of-package nutrition labeling on sugary beverage consumption and consequently on the prevalence of excess body weight and obesity and related direct costs in Brazil: An estimate through a modeling study
Source: PLoS One. 2023 Aug 11;18(8):e0289340. doi: 10.1371/journal.pone.0289340 (PMC10420370; doi:10.1371/journal.pone.0289340)
Supplement: S1 Table — (DOCX) [file pone.0289340.s010.docx]

S1 Table. Variables acquired from the VIGITEL database and used in the study.

| Variable | Variable description | Code |
| --- | --- | --- |
| Replica | Primary unit of analysis | NA |
| Year | Year VIGITEL was performed | NA |
| Q6 | Age (years) | NA |
| Q7 | Gender | 1 - male  2 - female |
| Q9 | Weight (kg) | 777 – does not know*  888 - does not know * |
| Q11 | Height (cm) | 777 - does not know *  888 - does not know * |
| Q14 | Pregnant | 1 - yes*  2 - no  777 - does not know |
| Q29 | How many days a week do you usually drink soft drinks or artificially flavored juice drinks? | 1 - 1 to 2 days a week  2 - 3 to 4 days a week  3 - 5 to 6 days a week  4 – Every day (including Saturday and Sunday)  5 – almost never*  6 - never* |
| Q30 | What type? | 1 - regular  2 - diet/ light/ zero*  3 - both |
| Q31 | How many glasses/cans do you usually drink per day? | 1 - glass/can per day  2 - glasses/cans per day  3 - glasses/cans per day  4 - glasses/cans per day  5 - glasses/cans per day  6 - 6 or more glasses/cans per day  7 – does not know* |
| Weight (through raking) | Expansion factor | NA |

NA: not applicable. *Participants who answered this code were excluded from this study.

More details are provided in the supporting information file (S1_File).
