# Supplementary material for: Impact of implementation of front-of-package nutrition labeling on sugary beverage consumption and consequently on the prevalence of excess body weight and obesity and related direct costs in Brazil: An estimate through a modeling study
Source: PLoS One. 2023 Aug 11;18(8):e0289340. doi: 10.1371/journal.pone.0289340 (PMC10420370; doi:10.1371/journal.pone.0289340)
Supplement: S2 Table — (DOCX) [file pone.0289340.s011.docx]

S2 Table – Profile of nutrients and their limits for liquid foods adopted by the Brazilian legislation.

| Nutrient per 100mL of liquids | Equal or higher quantity |
| --- | --- |
| Sodium (mg) | 100 |
| Added sugars (g) | 7.5 |
| Saturated fats (g) | 3 |

Source: Normative Instruction Nº75, Brazil (2020) [9].

More details are provided in the supporting information file (S1_File).
