# Supplementary material for: Impact of implementation of front-of-package nutrition labeling on sugary beverage consumption and consequently on the prevalence of excess body weight and obesity and related direct costs in Brazil: An estimate through a modeling study
Source: PLoS One. 2023 Aug 11;18(8):e0289340. doi: 10.1371/journal.pone.0289340 (PMC10420370; doi:10.1371/journal.pone.0289340)
Supplement: S3 Table — (DOCX) [file pone.0289340.s012.docx]

S3 Table – Profile of nutrients and their limits for liquid foods adopted by the Chilean legislation according to each implementation phase.

| Nutrient per 100mL of liquids | June 26 of 2016 | June 26 of 2018 | June 26 of 2019 |
| --- | --- | --- | --- |
| Energy (kcal) | 100 | 80 | 70 |
| Sodium (mg) | 100 | 100 | 100 |
| Total sugars (g) | 6 | 5 | 5 |
| Saturated fats (g) | 3 | 3 | 3 |

Source: Law 20,606, Chile (2012) [11].

More details are provided in the supporting information file (S1_File).
