# Supplementary material for: Impact of implementation of front-of-package nutrition labeling on sugary beverage consumption and consequently on the prevalence of excess body weight and obesity and related direct costs in Brazil: An estimate through a modeling study
Source: PLoS One. 2023 Aug 11;18(8):e0289340. doi: 10.1371/journal.pone.0289340 (PMC10420370; doi:10.1371/journal.pone.0289340)
Supplement: S4 Table — (DOCX) [file pone.0289340.s013.docx]

S4 Table – Beverages included in the study by Taillie et al. (2021).

| Sub-category | Description and examples |
| --- | --- |
| Soda | Carbonated soft drinks |
| Industrialized fruit and vegetable drinks | Industrialized fruit-flavored drinks, including powdered drinks, nectars, and ready-to-drink ones |
| Dairy-based beverages and dairy substitutes | Plain and flavored milks, dairy substitutes, and other dairy drinks, including powdered and ready-to-drink ones |
| Waters | Plain and flavored waters, mineral water, sparkling water, including powdered and ready-to-drink ones |
| Coffee and tea | Instant coffee, roasted coffee, ground coffee, RTD tea, and powdered tea |
| 100% fruit and vegetable juice | 100% fruit and vegetables juices, no additives |
| Sports drinks | Powdered and ready-to-drink sports drinks |
| Formula | Toddler/child formula, infant/baby formula, and maternal/elderly formula |

More details are provided in the supporting information file (S1_File).
