# Supplementary material for: Impact of implementation of front-of-package nutrition labeling on sugary beverage consumption and consequently on the prevalence of excess body weight and obesity and related direct costs in Brazil: An estimate through a modeling study
Source: PLoS One. 2023 Aug 11;18(8):e0289340. doi: 10.1371/journal.pone.0289340 (PMC10420370; doi:10.1371/journal.pone.0289340)
Supplement: S5 Table — (DOCX) [file pone.0289340.s014.docx]

S5 Table – Average differences in the purchase of beverages, in energy and sodium, before and after implementation of the policies observed by Taillie et al. (2021).

| Evaluated parameter | Beverages “high in” | Beverages without FoPNL | General beverage purchase |
| --- | --- | --- | --- |
| Energy - kcal/per capita/day (95%CI) | -16.3  (–18.7 to –13.9) | +6.3  (4.1 to 8.5) | -10.0  (–13.4; –6.6) |
| Energy (%) | -31.3 | +12.6 | -9.9* |
| Sodium - mg/per capita/day (95%CI) | -7.0  (–8.0 to –6.3) | +3.7  (1.6 to 5.9) | -3.4  (–5.8 to –1·1) |
| Sodium (%) | -43.3 | +7.6 | -5.2* |

*values used in modeling scenario 1 associated with the base scenario.

More details are provided in the supporting information file (S1_File).
