# Supplementary material for: Impact of implementation of front-of-package nutrition labeling on sugary beverage consumption and consequently on the prevalence of excess body weight and obesity and related direct costs in Brazil: An estimate through a modeling study
Source: PLoS One. 2023 Aug 11;18(8):e0289340. doi: 10.1371/journal.pone.0289340 (PMC10420370; doi:10.1371/journal.pone.0289340)
Supplement: S6 Table — (DOCX) [file pone.0289340.s015.docx]

S6 Table – Nutritional composition of beverages (median, interquartile range and average percentage changes) regarding the contents of energy and sodium during the pre-implementation period of the Chilean legislation observed by Kanter et al., (2019).

| Evaluated parameter | 2015 | 2016 | Average percentage changes |
| --- | --- | --- | --- |
| Energy - kcal/100mL | 30  (12, 44) | 28  (11, 44) | -1.6* |
| Sodium - mg/100mL | 10  (6, 17) | 10  (5, 17) | +1.8* |

*values used in modeling scenario 2 associated with scenario 1.

More details are provided in the supporting information file (S1_File).
