# Supplementary material for: Impact of implementation of front-of-package nutrition labeling on sugary beverage consumption and consequently on the prevalence of excess body weight and obesity and related direct costs in Brazil: An estimate through a modeling study
Source: PLoS One. 2023 Aug 11;18(8):e0289340. doi: 10.1371/journal.pone.0289340 (PMC10420370; doi:10.1371/journal.pone.0289340)
Supplement: S7 Table — (DOCX) [file pone.0289340.s016.docx]

S7 Table – Purchase of beverages observed in the experimental market study by Acton et al., (2019), in calorie and sodium average and percentage average variation, between the intervention and control groups.

| Beverage composition | Without labeling | “High in” labeling | Average variation (percentage) |
| --- | --- | --- | --- |
| Energy - kcal | 102.0 | 91.3 | -10.5* |
| Sodium – mg | 69.2 | 65.4 | -5.5* |

*values used in modeling scenario 3 for sensitivity analysis associated with base scenario.

More details are provided in the supporting information file (S1_File).
