# Supplementary material for: Impact of implementation of front-of-package nutrition labeling on sugary beverage consumption and consequently on the prevalence of excess body weight and obesity and related direct costs in Brazil: An estimate through a modeling study
Source: PLoS One. 2023 Aug 11;18(8):e0289340. doi: 10.1371/journal.pone.0289340 (PMC10420370; doi:10.1371/journal.pone.0289340)
Supplement: S8 Table — (DOCX) [file pone.0289340.s017.docx]

S8 Table – Estimations of Brazilian who consume sugary beverages and the prevalence of excess body weight and obesity up to 2024 based on the temporal trends and projections according to the VIGITEL survey.

| Temporal trends: | 2020  %  n | 2021  %  n | 2022  %  n | 2023  %  n | 2024  %  n |
| --- | --- | --- | --- | --- | --- |
| Sugary beverage consumers (1) | 45.3  54,043,044 | 44.5  53,447,776 | 43.7  52,747,104 | 42.9  52,004,688 | 42.1  52,203,903 |
| Excess body weight (2) | 60.1  32,469,061 | 61.4  32,816,934 | 62.7  33,082,983 | 64.0  33,303,802 | 65.4  34,120,471 |
| Obesity (3) | 22.8  12,294,793 | 23.6  12,597,641 | 24.4  12,859,744 | 25.2  13,099,981 | 26.0  13,573,015 |

More details are provided in the supporting information file (S1_File).
