# Supplementary material for: Impact of implementation of front-of-package nutrition labeling on sugary beverage consumption and consequently on the prevalence of excess body weight and obesity and related direct costs in Brazil: An estimate through a modeling study
Source: PLoS One. 2023 Aug 11;18(8):e0289340. doi: 10.1371/journal.pone.0289340 (PMC10420370; doi:10.1371/journal.pone.0289340)
Supplement: S9 Table — (DOCX) [file pone.0289340.s018.docx]

S9 Table – Estimations of the prevalence of excess body weight and obesity in Brazilian consumers of sugary beverages after the implementation of front-of-package labeling up to 2024, and sensitivity analysis.

| Estimated prevalence | 2020  % | 2021  % | 2022  % | 2023  % | 2024  % |
| --- | --- | --- | --- | --- | --- |
| Scenario 1  Obesity  Excess weight | 22.3  59.6 | 22.8  60.4 | 23.9  61.8 | 24.5  63.2 | 25.3  64.4 |
| Scenario 2  Obesity  Excess weight | 22.2  59.5 | 22.7  60.4 | 23.6  61.7 | 24.4  63.0 | 25.2  64.2 |
| Scenario 3  Obesity  Excess weight | 22.2  59.5 | 22.8  60.4 | 23.7  61.7 | 24.4  63.0 | 25.2  64.3 |
| Scenario 4  Obesity  Excess weight | 22.2  59.5 | 22.7  60.3 | 23.4  61.6 | 24.4  62.9 | 25.1  62.2 |

More details are provided in the supporting information file (S1_File).
