# Supplementary material for: Impact of implementation of front-of-package nutrition labeling on sugary beverage consumption and consequently on the prevalence of excess body weight and obesity and related direct costs in Brazil: An estimate through a modeling study
Source: PLoS One. 2023 Aug 11;18(8):e0289340. doi: 10.1371/journal.pone.0289340 (PMC10420370; doi:10.1371/journal.pone.0289340)
Supplement: S10 Table — (DOCX) [file pone.0289340.s019.docx]

S10 Table – Estimations of the reduction in prevalence of excess body weight and obesity in the Brazilian population attributed to the implementation of the front-of-package up to 2024, and sensitivity analysis.

| Reduction in prevalence | 2020  pp | 2021  pp | 2022  pp | 2023  pp | 2024  pp |
| --- | --- | --- | --- | --- | --- |
| Scenario 1  Obesity  Excess weight | -0.19  -0.22 | -0.35  -0.42 | -0.21  -0.41 | -0.29  -0.36 | -0.32  -0.42 |
| Scenario 2  Obesity  Excess weight | -0.25  -0.26 | -0.37  -0.45 | -0.32  -0.44 | -0.35  -0.45 | -0.35  -0.48 |
| Scenario 3  Obesity  Excess weight | -0.25  -0.25 | -0.35  -0.44 | -0.29  -0.43 | -0.34  -0.42 | -0.32  -0.46 |
| Scenario 4  Obesity  Excess weight | -0.25  -0.27 | -0.37  -0.49 | -0.34  -0.47 | -0.35  -0.47 | -0.37  -0.48 |

More details are provided in the supporting information file (S1_File).
