# Supplementary material for: Impact of implementation of front-of-package nutrition labeling on sugary beverage consumption and consequently on the prevalence of excess body weight and obesity and related direct costs in Brazil: An estimate through a modeling study
Source: PLoS One. 2023 Aug 11;18(8):e0289340. doi: 10.1371/journal.pone.0289340 (PMC10420370; doi:10.1371/journal.pone.0289340)
Supplement: S11 Table — (DOCX) [file pone.0289340.s020.docx]

S11 Table – Estimations of the direct costs regarding obesity according to what Nilson et al. described for the total Brazilian population and for the age group of 20 to 59 years.

| Costs | 2018  (20-59 years) | 2018  (per capita) | 2018  obesity cases assisted by SUS (n) |
| --- | --- | --- | --- |
| Direct costs  (reais - R$) | 725,568,380.76 | 43.38 | 16,724,223 |
| Direct costs  (American dollar*) | 187,243,453.10 | 11.20 |  |

*Exchange rate in 2018: R$ 3.875.

More details are provided in the supporting information file (S1_File).
