# Supplementary material for: Impact of implementation of front-of-package nutrition labeling on sugary beverage consumption and consequently on the prevalence of excess body weight and obesity and related direct costs in Brazil: An estimate through a modeling study
Source: PLoS One. 2023 Aug 11;18(8):e0289340. doi: 10.1371/journal.pone.0289340 (PMC10420370; doi:10.1371/journal.pone.0289340)
Supplement: Attachment — Submitted filename: Response to Reviewers.docx [file pone.0289340.s021.docx]

Dear Authors,

This paper on 'Impact of the implementation of front-of-package nutrition labeling on sugary beverage consumption on overweight and obesity and related direct costs in Brazil: a modeling study' has the potential to contribute to an important area of public health, given the challenge of tackling the obesity epidemic. There are, however, several issues that must be addressed by the authors which have been noted by the Reviewers.

Dear Editor,

We appreciate the opportunity to review and improve our article. We would like to thank the reviewers for their time and essential comments. In this review, we address the reviewers' suggestions and the important comments of the Ph.D. qualifying exam committee of the Ph.D. student, Natália C. Faria. They have suggested improvements in the experimental data - as we now have real evidence of FoPNL on purchase from Chile (and this is also a closer population about Brazil than Canadians). So, we have also changed the experimental source of data too.

The title needs to clarify that the Impact is a **'projection or an estimate**', rather than an evaluation of an established intervention.

We rewrote the title, including your suggestion: Impact of the implementation of front-of-package nutrition labeling on sugary beverage consumption on excess body weight and obesity and related direct costs in Brazil: **an estimate through** a modeling study.

There appear to be concerns noted by the authors regarding data access and this is potentially a cause for concern.

We agreed and removed the part of the data not shown and adjusted the work method. We entered these data as part of the sensitivity analysis described in the work method and with a detailed table in a supplementary file.

Line 291: “Sensitivity analysis:

Line 308: The range of uncertainty regarding the impact of FoPNL implementation on the prevalence of obesity and excess body weight was determined using sensitivity analysis. For this purpose, alternative scenarios were simulated. The scenario 3 impact promoted by FoPNL in adults (n = 1,213) regarding the purchase of sweetened beverages observed in the Canadian experimental market study (energy: −10.5%; sodium: −57.56%) was used [40,41], which was consistent with the trend of consumption of sugary beverages in the Brazilian population. Acton et al. obtained their results through an experimental market study conducted in Canada using a “high in” FoPNL in a red circle [40]. Finally, scenario 4 was based on reformulation of beverages (energy: −1.6%; sodium: 1.8%) observed by Kanter et al. in Chile [14], and was considered to be associated with scenario 3 described above.

Additionally, **the Ethics approval dates from 2013, and it would be helpful to provide more up-to-date approval.**

Dear Editor, we appreciate your comment. The survey VIGITEL was approved by the National Commission of Ethics in Research for Human Beings of the Ministry of Health (CONEP Opinion 355.590 of 26 June 2013 and certificate of presentation for ethics assessment - CAAE - number 16202813.2.0000.0008). It is mentioned as it is in many other papers that used this database, like:

https://doi.org/10.1590/1516-3180.2017.0044250517

https://doi.org/10.1186/s12939-021-01533-z

https://doi.org/10.1007/s10433-021-00659-x

<https://doi.org/10.1590/S1679-49742021000100008>

The Abstract does not coherently present the **rationale, methods, results nor conclusion** of the study, and like the rest of the paper, would benefit from an editorial revision by an expert English-speaker. This has been noted by Reviewer 1.

We appreciate the editor's comments about our paper. We have now included the sections mentioned on the Abstract. Our paper has passed through a native English speaker review.

The Introduction is also verbose and **needs to be precise, succinct and focused.** The justification for this study as presented in lines 63-65 is weak, and at odds with the subsequent text of lines 85-87.

We thank the editor for this important observation. We have included some important elements that reviewer 2 asked us for and removed some text to improve the justification and precision. We also tried to improve the justification of the study.

The term **'direct costs' is not reviewed in the Background,** nor is the justification for this evaluation made clear by the Authors. Appropriate information must be provided, and the specific **Methods used to calculate such costs**, must be clearly presented with supporting justification in the relevant section.

We appreciate the important observation and have included the term direct costs in the background and justification of the study. We also created a supplementary file to explain in more detail the methods developed in work.

In the Abstract, line 33: “Rationale: Intake of sugary beverages has been associated with obesity and chronic non-communicable diseases, thereby increasing the direct health costs related to these diseases.”

And in the introduction, in line 80: “Obesity and its effects on health generated an estimate annual costs of R$ 1.42 billion (95% CI, 0.98–1.87) in 2018 via the Public Health System (SUS) in Brazil [8]. The costs included hospitalizations, outpatient procedures, and medications distributed by the SUS for the treatment of these diseases, excluding supplementary health costs in the country, as well as the economic and social costs associated with illness and death from these causes [8]. Direct costs are those paid by health services related to immediate expenses, and include labor, tests, and medications [9].”

There is also **no discussion about the relevance, impact or calculation of the impact of Beverage reformulation in the Introduction, yet this is also a key indicator**. Furthermore, the evaluation of the impact of FoPNL is combined with Beverage reformulation in the analyses without clear justification, nor presentation of the specific analyses used.

We thank you and agree with the suggestion. The relevance of reformulating beverages was included in the introduction, as shown in the excerpt below. In the supplementary file we show more details of the analyzes used.

In the Introduction, line 99: “In Chile, the food industry reformulated foods and beverages after implementing FoPNL and other public health policies, reducing added sugars in sugary beverages and introducing non-nutritive sweeteners [14,15]. Kanter et al. found a reduction in the content of added sugars in beverages between 2015 and 2016 (median 7.5 g/100 mL, interquartile range (IQR): 2.3–10.0 in 2015 to 6.0 g/100 mL, IQR: 2.2–10 in 2016) in Chile, referring to reformulation [14].”

Statements such as **'Estimated from the perspective of the public health system (line 112) must be clearly explained**.

We appreciate the suggestion and expand on the explanation as described below:

In the Method, line 154: “Additionally, the reduction in direct costs related to obesity and excess body weight in the Brazilian population was estimated from the perspective of the public health system. In Brazil, a significant portion of the population depends on SUS, representing 71.5% of Brazilians who do not have access to supplementary health services [31]. Most SUS expenses related to disease treatment are based on outpatient procedures, hospital care, and dispensing of medicines to control chronic diseases by the Popular Pharmacy Program [32].”

The **Methodology used to Estimate energy intake based on use of Vigitel data between 2007 and 2019 (except for 2017) is not clear.** The use of linear regression gives the sense that relevant statistical analyses were used, but the Methodology must be presented (referenced relevant formulae) and what it actually means. **The lack of data for 2017 must also** be explained as its implications for the subsequent estimates.

We agree and have included explanations in the method of work and supplementary file. In calculating the tendency to consume sugary beverages in the Brazilian population, database from 12 years reported by individuals Brazilians in the VIGITEL survey were analyzed. However, the question analyzed for calculating the consumption trend in the Vigitel survey differed in 2017 compared to the other years analyzed. In 2017, the question referred to the amount of consumption of sugary beverages on the day before the survey, while in other years, it was related to the usual amount when consuming sugary beverages. As they are different questions, the year 2017 was excluded. This explanation was included in the supplementary material developed.

In the Method, line 285: “The analyzed population was described according to characteristics related to sex and age and presented as the mean and 95% confidence interval (95% CI). The prevalence of sugary beverage consumers and their average daily consumption, stratified by sex, were estimated for each year using the VIGITEL survey. The consumption of sugary beverages was presented as the average and 95% CI. Linear trends were investigated using linear regression analysis. The dependent variables evaluated for each year included the annual proportion of Brazilians who consumed sugary beverages, average amount of sugary beverages consumed (in terms of energy and sodium), annual proportion of obese Brazilians who consumed sugary beverages, and annual proportion of Brazilians with excess body weight who consumed sugary beverages. The independent variables included data of the VIGITEL survey or all years used in this study. Regression models were stratified by sex, considering the sample weights of the VIGITEL survey. Thus, temporal trends (2007–2019, except 2017) were analyzed, and the projected prevalence (2020–2024) was calculated in the base scenario. Additional details are provided in the supplementary file.”

**The meaning of 'soft drinks or artificial juices' needs to be explained in the national context**, so the reader can understand the actual beverages captured by this term. Has there been any independent validation of this indicator?

We thank the reviewer for the important observation. A definition of soft drinks and artificial juices according brazilian regulations were included in the text. Line 196: “According to the Technical Regulation in Brazil, soft drinks are carbonated beverages obtained by dissolving the juice or plant extract in drinking water, added sugar, and saturated with industrially pure carbon dioxide [35]. In this study, only soft drinks were considered as sugary beverages.”

There was a validation and reproducibility analysis carried out in random sub-samples (n=112 and n=109) of the total number of participants (N=2,204) in adults aged 18 years or older, in 2005, of the VIGITEL survey. Among the evaluated indicators were the evaluation of the daily or almost daily consumption of soft drinks. For reproducibility analysis, the results obtained in the original telephone interview were compared with results obtained in another telephone interview carried out between seven and 15 days after the original interview. Kappa coefficients indicated substantial agreement (all: 0.77; men: 0.83; women: 0.72). For validity analysis, the results of the telephone interview were compared with three 24-hour recalls carried out 15 days after the original interview. Reasonable values of specificity (all: 94.1%; men: 93.3%; women: 94.6%) and sensitivity (all: 87.5%; men: 50%; women: 100%) were observed for soft drink consumption.

Details are described in this publication:

https://doi.org/10.1590/S0034-89102008000400002

We included this information in the method (line 264) and supplementary file.

Line 193: “The questions related to soft drink consumption used in the VIGITEL survey passed through a previous validation analysis with reasonable values of specificity (all: 94.1%) and sensitivity (all: 87.5%) [34].”

Although this is an important question, all the sugary beverage consumption was considered as “soft drink” consumption based on the nutritional composition of this beverage.

Line 157 speaks to 'The amount of energy (kcal/day) from sugary drinks was calculated based on the nutritional table [29], using standardized procedures'. **Please explain these 'standardized procedures**'...

We appreciate the suggestion and add in these procedures to a method.

In the Method, line 200: The average consumption of sugary beverages (mL/day) was estimated conform described in a previous study [7]. The average consumption volume was defined as 250 mL to represent the average value between a glass (150 mL) and a can (350 mL) of soda based on the VIGITEL data. The amount of calorie (kcal/day) intake from sugary beverages was based on the Brazilian Table of Food Composition [36]. Further details can be found in the supplementary file.”

The sections on 'Estimation of energy intake reduction, Estimated reduction in body weight, BMI, and prevalence of obesity and overweight, Reduction in obesity and overweight cases, Cost estimation - **each need to clearly present the Methods, Formulae used, as well as Justification for the analytical methods used, in a comprehensive and transparent way, that allows the non-expert reader to follow the process.** The Methods must be understandable, and this Section must be improved.

We appreciate the suggestion and have created a supplemental file to detail the methods used.

**Line 254: Do these Methods consider the appreciation of the real value of money over time? Do the estimates based on the work of Oliveira et al (2011) still have relevance in 2022?.**

We appreciate the suggestion and decided to change the cost baseline study. We replaced it with the study by Nilson et al., 2020 (https://doi.org/10.26633/RPSP.2020.32). This study describes the direct costs attributable to obesity, hypertension and diabetes in Brazil's unified health system (SUS) in the year (2018). Thus, the value update referred to only one year.

The Authors also speak to cost savings of $3.5 million over 5 years (line 507); is this the total **savings nationally, in the public service or in other sectors**??

This possible economy will be at the national level, for the single health system in Brazil. We made it clearer in line 398.

**The Discussion uses 9 pages of text and can be considerably shortened** if the Authors present their case precisely and succinctly.

Please note the issues highlighted particularly by Referee 1.

We agree and completely reframe the discussion.

[Note: HTML markup is below. Please do not edit.]

Reviewers' comments:

Reviewer's Responses to Questions

**Comments to the Author**

1. Is the manuscript technically sound, and do the data support the conclusions?

The manuscript must describe a technically sound piece of scientific research with data that supports the conclusions. Experiments must have been conducted rigorously, with appropriate controls, replication, and sample sizes. The conclusions must be drawn appropriately based on the data presented.

Reviewer #1: Partly

Reviewer #2: Yes

2. Has the statistical analysis been performed appropriately and rigorously?

Reviewer #1: Yes

Reviewer #2: I Don't Know

3. Have the authors made all data underlying the findings in their manuscript fully available?

The [PLOS Data policy](http://www.plosone.org/static/policies.action#sharing) requires authors to make all data underlying the findings described in their manuscript fully available without restriction, with rare exception (please refer to the Data Availability Statement in the manuscript PDF file). The data should be provided as part of the manuscript or its supporting information, or deposited to a public repository. For example, in addition to summary statistics, the data points behind means, medians and variance measures should be available. If there are restrictions on publicly sharing data—e.g. participant privacy or use of data from a third party—those must be specified.

Reviewer #1: Yes

Reviewer #2: Yes

4. Is the manuscript presented in an intelligible fashion and written in standard English?

PLOS ONE does not copyedit accepted manuscripts, so the language in submitted articles must be clear, correct, and unambiguous. Any typographical or grammatical errors should be corrected at revision, so please note any specific errors here.

Reviewer #1: No

Reviewer #2: Yes

5. Review Comments to the Author

Please use the space provided to explain your answers to the questions above. You may also include additional comments for the author, including concerns about dual publication, research ethics, or publication ethics. (Please upload your review as an attachment if it exceeds 20,000 characters)

Reviewer #1: Overall comments: The paper gives useful information although it is a descriptive level analysis that is presented. **There is too much repetition throughout the paper that needs to be addressed**, and it would benefit from giving more information about the actual models used than just referencing the models that the simulations were based on. Overall, there is merit to the study. While the grammar in the overall paper is acceptable, the abstract needs proofreading.

We appreciate the opportunity and have reviewed the entire article. We have created a supplemental file to detail the methods of the work.

The abstract needs reworking, considering the below:

Line 2 should be more impactful as using ‘may” hints at uncertainty. “Intake of sugary beverages has been associated with CNCDS…”

We agree and adjust the text.

The abstract and main body of the paper do not align in terms of whether the FoPNL has actually been implemented in Brazil. Clarity is needed and changes to with the abstract or main paper made to align.

We apologize for this and have revised the text. The legislation passed in October 9th, 2020 and implementation of FoPNL in Brazil started on October 9th, 2022.

Abstract needs to be proofread for grammar, for example in the abstract line 7 “A simulation study...” line 10 “The following scenarios…” line 14 Change in body weight was estimated by a simulation model conducted by Hall etc al.” etc.

We apologize and pass all work through a review.

The scenarios in the abstract are not clear as to what is actually being simulated—it is clearer in the paper methods (line 99) what the three scenarios are.

We are sorry for that. Now, the scenarios in the abstract are described as (line 43):

“The following scenarios were considered: base (trend in sugary beverage intake); 1 (base scenario associated with the changes in energy content of the purchased beverages observed after the first phase of the Chilean labeling law (−9.9%); and 2 (scenario 1 associated with reformulation of beverages, total energy reduction of −1.6%).”

What does line 15 “Linear trend…” mean in terms of the simulation?

Linear regression was used to analyze the temporal trend prevalence of obesity/excess body weight in Brazilian population. We adjust the text:

In abstract, line 48: “A linear trend in the prevalence of obesity and excess body weight in the Brazilian population was considered.”

Main paper comments:

Need references for line 63-65.

We appreciate your note and have included the references (line 177):

doi:10.3389/fnut.2022.898021

doi:10.3389/fnut.2022.921065

doi:10.1371/journal.pone.0265990

Line 82, give the direction of the impact on the number of obese people in the country.

We agree with the suggestion and adjust the text, as follows:

Line 118: “A modeling study conducted in Mexico estimated that FoPNL can promote a reduction of 23.2 kcal/day (95% CI, −24.5 to −21.9) associated with the consumption of sugary drinks and 13.6 kcal/day (95% CI, −14.1 to −13.1) to snacks, resulting in 4.98pp reduction in the number of obese people in the country [26].”

Line 90, specify direct costs to whom (i.e. to the public health system).

We appreciate the suggestion and have included it in the text, as described below:

Line 131: “Thus, the objective of this study was to estimate, over five years, the reduction in the prevalence of obesity and excess body weight among Brazilian adults and the direct costs in public health system related to such problems after the implementation of FoPNL in Brazil.”

Describe what direct costs to the public health system are.

We complete the text, as below, in the introduction::

Line 80: “Obesity and its effects on health generated an estimate annual costs of R$ 1.42 billion (95% CI, 0.98–1.87) in 2018 via the Public Health System (SUS) in Brazil [8]. The costs included hospitalizations, outpatient procedures, and medications distributed by the SUS for the treatment of these diseases, excluding supplementary health costs in the country, as well as the economic and social costs associated with illness and death from these causes [8]. Direct costs are those paid by health services related to immediate expenses, and include labor, tests, and medications [9].

In the scenarios detailed from line 99, **what is the definition of reformulation for the purposes of this study which is considered in scenario 3**? The literature reviewed in the introduction do not speak to the impact of reformulation and it should be included if it is being used in scenario 3. This can be addressed by moving sections of the discussion to the introduction

We appreciate the important note and have included the excerpt below about the recast in the introduction.

Line 99: “In Chile, the food industry reformulated foods and beverages after implementing FoPNL and other public health policies, reducing added sugars in sugary beverages and introducing non-nutritive sweeteners [14,15]. Kanter et al. found a reduction in the content of added sugars in beverages between 2015 and 2016 (median 7.5 g/100 mL, interquartile range (IQR): 2.3–10.0 in 2015 to 6.0 g/100 mL, IQR: 2.2–10 in 2016) in Chile, referring to reformulation [14].”

What was the reason for excluding persons with extreme BMI (line 134)?

There is a possibility that these people have atypical habits in food consumption, so to standardize we exclude the extremes. The same procedure was performed in another study:

https://doi.org/10.1371/journal.pmed.1003221

Why was the year 2017 excluded (line 164)?

To calculate the tendency of consumption of sugary beverages in the Brazilian population, data from 12 years reported by Brazilians in the Vigitel survey were analyzed. However, the question analyzed for calculating the consumption trend in the Vigitel survey was different in 2017 compared to the other years analyzed. In 2017, the question refers to the amount of consumption of sugary drinks on the day before the survey, while in other years, it is related to the usual amount when consuming sugary drinks. As they are different questions, the year 2017 was excluded. This explanation was included in the supplementary material developed.

Line 240 is unclear “…including the one…” –so were there multiple studies and not just one study? The study needs a bit more description for clarity.

We have changed the base article used for the cost analysis. The topic Cost estimation in method was rewritten.

Lines 246 to 252 have repetition regarding how obese individuals were calculated, but also does not detail exactly how this subset was determined.

The estimate of the number of obesity cases was obtained by the difference between scenarios 1 and 2 with the temporal trend of obesity prevalence. We have added more information in the supplementary material.

I don not think lines 288-294 or 316-322 or 335-341 or 362-367 are necessary under the tables/figures as they are repeating what has already been described in the methods. Instead the word count saved by taking those lines out can be used to describe in more detail the simulation models that were used as the variables being put into those models were described, but the actual models were referenced.

We appreciate the suggestion and are removing the suggested text.

The discussion section needs to be revised, consider the below comments:

Lines 382-406 are better suited for the introduction.

We appreciate the suggestion and have completely revamped the discussion.

Line 408-412 is unclear, in the introduction it may help to give more details about the Acton study since it is mentioned repeatedly in this paper.

We chose to remove this section because we changed the base study of the modeling.

Again, lines 414-26 should be in the intro, with line 427-428 then referenced as appropriate within the discussion.

Lines 463-474 also could be part of the introduction.

We appreciate the suggestion and move the text to the introduction section.

How does the sentence in lines 484-486 related to the findings of your study? If a different model is adopted in Brazil would better outcomes be expected?

Evidence on the effect that the black magnifying glass design's effect on the population is still scarce. Studies show that, depending on the FoPNL design adopted, different effects are observed. As the black magnifying glass design was adopted in Brazil, with a different cut-off point for critical nutrients adopted in Chile, the results may be different than expected. However, the scenario based on the Chilean study was adopted because it is an evaluation after the implementation of public policy, in addition to considering some compensation in the consumption of beverages (reduction in purchases of “high em” beverages and increase in purchases of beverages without FoPNL), relevant points for the methodological choice.

We have now included the differences between our labeling system and the Chilean on the supplementary material to a better understanding.

Line 507, what percentage of the costs to public health is US$3.5 million? Is the reduction in cost significant?

This reduction represents 2.8% of the total health costs with obesity in the 20-59 age group, adjusted for 2024 (with updated cost estimates based on latest database work – US$5.5 millions).

The overall impact of the simulation is not clear. Is the reduction in obesity impactful as compared to other simulation studies? Is the reduction in cost impactful compared to other studies? While some studies have been referenced, the utility of this study in comparison has not been adequately discussed.

This study shows that the implementation of FoPNL in Brazil has the potential to slow down the increase in the prevalence of obesity and overweight in the Brazilian population that consumes sugary drinks. FoPNL represents one of the public policies that can be used as an obesity prevention strategy. Due to the vast majority of Brazilians being dependent on public health services, the reduction in the prevalence of obesity and, consequently, of CNCDs has the potential to reduce the burden on the system and thus impacts on the reduction of its costs. Bastos-Abreu et al. (2020) also projected the impact of FoPNL implementation in the Mexican population. However, it did not show a temporal trend in the prevalence of obesity. Also, this study and Bastos-Abreu et al. corroborate the results of the reduction of cases of obesity.

Reviewer #2: 179-181: I don't understand this passage. Is this downward trend in Brazil related to the use of FOPNLs? How is this result connected to the one we see in Canada after using a FOPNL?

We reviewed the study and considered the study by Taillie et al. (2021), which evaluated the effect after the first phase of the implementation of Chilean legislation. As there is some compensation in the consumption of beverages and because the policy is being implemented, we believe the results can be closer to what can happen in Brazil.

Conclusion: I honestly don't see how a reduction of 26 kcal/die and a shift from a BMI of 26.5 kg/m2 to 26.1 kg/m2 in 5 years, with a weight reduction of 1 kg, can be seen as a "potential to reduce overweight and obesity prevalence". Honestly, it also seems to me too big a leap to go from these results to saying that FOPNLs may lead to a reduction in NCDs.

Dear reviewer, we thank you for your important comment. In fact, simulation studies are done worldwide the way we have performed. We agree and point out that the FoPNL implementation strategy can help to prevent cases of obesity and overweight and slow down your increase, as described in the last paragraph, copied below:

Line 524: “In conclusion, a reduction in energy intake is estimated along with a consequent reduction in the prevalence of obesity by 0.32pp after five years of FoPNL implementation in Brazil. This reduction in obesity prevalence may save US$ 5,5 million (95% CI 4,7 to 8,8) in the public budget. This amount may even be invested in the promotion of physical activity, educational advertisements, and actions aimed at schools. Implementing FoPNL can improve consumer understanding of the nutritional value of a product, making product choice easier. FoPNL has the potential to collaborate in the treatment of obesity, although further actions are necessary to prevent the occurrence of obesity/excess body weight and thus reduce related public health costs.”

Although the impact of energy reduction is modest, it is worth mentioning that, in this study, the effect of implementing FoPNL was modeled in a single product category, which are sugary beverages (soft drinks). Other studies have modeled modest reductions in energy intake and shown benefits in the population, such as those highlighted below:

<https://doi.org/10.1371/journal.pmed.1003221>

https://doi.org/10.1016/j.amepre.2019.06.022

<https://doi.org/10.1038/ijo.2010.228>

https://doi.org/10.1186/s12966-019-0817-2,

This category of beverages, is consumed by almost half of the Brazilian adults residing in Brazilian capitals. In addition, other public health policies can be implemented, such as taxation and restrictions on product marketing, as well as food and nutrition education actions in the population to intensify obesity prevention.

Even if it's true that the public health system can benefit from the reduction in obesity cases, I don't think we can say that FOPNLs alone can achieve that. And since this kind of studies will be used from the policymakers in the near future to implement health policies, I believe it **is necessary to always dedicate a relevant section of the papers to the need to implement education policies at a national level to push people to make better choices all round, and not just by comparing (assuming they do) labels at the supermarket**.

We agree with this and highlight in the last paragraph the need to implement other strategies to prevent obesity (line 527). In addition, in the discussion, we highlight that in Chile, other actions were developed, such as restrictions on marketing and sales in schools, as well as taxation, to work with the implementation of FoPNL (line 503).
