# Supplementary material for: Impact of implementation of front-of-package nutrition labeling on sugary beverage consumption and consequently on the prevalence of excess body weight and obesity and related direct costs in Brazil: An estimate through a modeling study
Source: PLoS One. 2023 Aug 11;18(8):e0289340. doi: 10.1371/journal.pone.0289340 (PMC10420370; doi:10.1371/journal.pone.0289340)
Supplement: Attachment — Submitted filename: Response to reviewers2.docx [file pone.0289340.s022.docx]

**Response to reviewers**

Journal Requirements:

Please review your reference list to ensure that it is complete and correct. If you have cited papers that have been retracted, please include the rationale for doing so in the manuscript text, or remove these references and replace them with relevant current references. Any changes to the reference list should be mentioned in the rebuttal letter that accompanies your revised manuscript. If you need to cite a retracted article, indicate the article’s retracted status in the References list and also include a citation and full reference for the retraction notice.

We’ve reviewed the references.

Additional Editor Comments:

While many of the issues have been addressed, there are a number of editorial issues highlighted by a referee which need to be addressed.

We appreciate the opportunity to review this paper again, and we answered to all of the questions listed by the reviewer.

We want to emphasize that reviewer #3 probably reviewed the first version we submitted. We wonder if this happened because he/she asked for suggestions we had already addressed. Also, the number of the lines he/she mentioned were not the same as the last reviewed paper.

Of note a concern has also been raised about the importance of sensitivity analyses around the estimates made using statistical modeling techniques, and it would be important for the authors to respond to this.

We appreciate the reviewer's suggestions and have answered the question about sensitivity analysis below. Sensitivity analysis was included in the first revision.

Reviewers' comments:

Reviewer's Responses to Questions

Comments to the Author

1. If the authors have adequately addressed your comments raised in a previous round of review and you feel that this manuscript is now acceptable for publication, you may indicate that here to bypass the “Comments to the Author” section, enter your conflict of interest statement in the “Confidential to Editor” section, and submit your "Accept" recommendation.

Reviewer #2: All comments have been addressed

Reviewer #3: (No Response)

2. Is the manuscript technically sound, and do the data support the conclusions?

The manuscript must describe a technically sound piece of scientific research with data that supports the conclusions. Experiments must have been conducted rigorously, with appropriate controls, replication, and sample sizes. The conclusions must be drawn appropriately based on the data presented.

Reviewer #2: Yes

Reviewer #3: Partly

3. Has the statistical analysis been performed appropriately and rigorously?

Reviewer #2: I Don't Know

Reviewer #3: I Don't Know

4. Have the authors made all data underlying the findings in their manuscript fully available? The PLOS Data policy requires authors to make all data underlying the findings described in their manuscript fully available without restriction, with rare exception (please refer to the Data Availability Statement in the manuscript PDF file). The data should be provided as part of the manuscript or its supporting information, or deposited to a public repository. For example, in addition to summary statistics, the data points behind means, medians and variance measures should be available. If there are restrictions on publicly sharing data—e.g. participant privacy or use of data from a third party—those must be specified.

Reviewer #2: Yes

Reviewer #3: Yes

5. Is the manuscript presented in an intelligible fashion and written in standard English? PLOS ONE does not copyedit accepted manuscripts, so the language in submitted articles must be clear, correct, and unambiguous. Any typographical or grammatical errors should be corrected at revision, so please note any specific errors here.

Reviewer #2: Yes

Reviewer #3: Yes

6. Review Comments to the Author

Please use the space provided to explain your answers to the questions above. You may also include additional comments for the author, including concerns about dual publication, research ethics, or publication ethics. (Please upload your review as an attachment if it exceeds 20,000 characters)

Reviewer #2: (No Response)

Reviewer #3: Abstract

Dear Reviewer, we appreciate your essential contributions to our manuscript and the opportunity to answer to your questions. The version you used to send us suggestions has already gone through a first review. Therefore, we added the responses to the last version of the article.

Line 3: The word nutrition should be inserted before labelling so that the meaning of the abbreviation is clear.

There are several places in abstract where grammar/syntax is compromised presumably to conserve words. This should not be done as it makes the text less clear.

We included the word “nutrition” in the requested line. Thank you for your observation (line 35).

Line 5: To estimate, over five years, THE impact of implementing

We thank you and adapted the sentence as suggested (lines 37-38).

Line 7: A Simulation study to

We adapted the text as proposed. Thank you for that (line 40).

Line 9: The VIGITEL research

Line 9: VIGITEL research database (2019)- do you mean the 2019 version or data for the year 2019? That is unclear.

We used data collected in 2019 and published in the 2020 report. As requested, we have adapted the text to be clearer (line 43).

Line 9: Simply saying “n” is insufficient. Should say sample size if that is what n stands for.

We included this explanation for clarity in lines 43-44: (the final sample consisted of 12,471 data points representing 14,380,032 Brazilians).

Line 10: Instead of “Following scenarios were considered” could be “The scenarios considered were:”

We appreciate the suggestion and changed the text accordingly (line 45).

Line 19: “CI95%” - should be 95% CI

We apologize for this mistyping and we have revised all of the text to 95% CI.

Line 23: the abbreviation “pp” is unclear to me. If it stands for ‘percentage points’ which I am assuming/guessing from the main text then this should be clearer from the abstract.

We removed the abbreviation and used “percentage points” instead to improve understanding (lines 60-62).

Introduction

Lines 64-65:

“However, even though the impact of FoPNL on food purchase

64 and consumption in the Brazilian population has been investigated, it still needs

65 further studies.”

Recommendation: Authors could indicate here what further investigations are needed. The statement as stands in vague. Given their study, it could read further investigations needed to understand how consumption has impacted obesity rates etc.

We added the sentence below, on lines 132-135:

“In addition, investigations are needed to understand consumer behavior because of the implementation of FoPNL, such as possible changes in purchasing patterns, consequent changes in food consumption and causing changes in nutritional status.”

Lines 66-70 references a meta-analysis but not the population/country where done. But country is mentioned for other studies in the same and ensuing paragraphs. Feels inconsistent.

We appreciate the suggestion and completed the text. Individuals included are Canadians and Americans; this information was included in line 101 as requested: (individuals Canadians and Americans were included in the analysis).

Methods

Generally very well explained.

We appreciate the comment.

I found the system of referring to base scenario then scenario 1 and scenario 2 somewhat confusing. Maybe rename so scenario 1 is base scenario or base scenario could be 0.

Thanks for the suggestion; however, we are concerned that Scenario 0 may be understood as a “business-as-usual” or baseline scenario, in which nothing has been done. We followed the time trend observed in the consumption of sugary beverages, as well as the time trend in the prevalence of obesity, both according to the adjustments used in the study, and therefore we chose to keep it as the baseline scenario, as it refers to what comes next. occurring in recent years. We apologize and ask that you consider keeping the base scenario name.

Line 117- “database for 2019”. Does that mean the version released in 2019 or data for the year 2019?

This means that data was collected in 2019 and published in the report in 2020. We apologize for not being transparent and have adjusted the text on lines 164-166: The VIGITEL (Surveillance System for Risk and Protective Factors for Chronic Diseases by Telephone Survey) database 2019 and published in the 2020 report was used to conduct the study.

Line 193: The authors state: The effect of reducing energy intake of sugary drinks from changes in FoPNL disregarded saturated fats and sugars to estimate body weight variation. Can the authors explain how this might have under or overestimated the overall effects of FOPNL on obesity prevalence?

In the VIGITEL survey, the volunteer is asked about consuming soft drinks or artificial juices, using soft drinks as the standard caloric value. In the mathematical model, the delta used refers to energy. Other sugary drinks may have fat in their composition, but we did not consider them in our analyses. We agree that this is a limitation of our study and included a sentence about it in lines 535-538: “Furthermore, another limitation refers to the use of soda as the only sugary beverage in this study. Other sugary beverages may contain fats in their composition, which were not considered in this analysis, which could affect the results obtained in the modeling.” We appreciate this observation.

Major: In modelling studies, given the nature of simulations and predictions it is best practice to run sensitivity analyses around the assumptions made. This is a major omission from this paper. The authors chose Acton et al for estimating the effect of the FOPNL on SS consumption. The reason is sound but given that a meta-analysis exists, the findings from the meta-analysis and/or studies from the analysis should be used as part of a sensitivity analysis in the model to determine the extent of the difference had a different paper/estimate been chosen.

We agree with your observation and we included a sensitivity analysis after the first review, described in Appendix. There were changes in the modeled scenarios, and we chose to use the evaluation of the policy implemented in Chile through the results of the study of Taillie LS, Bercholz M, Popkin B, Reyes M, Colchero MA, Corvalán C. - Changes in food purchases after the Chilean policies on food labelling, marketing, and sales in schools: a before and after study. Lancet Planet Heal. 2021;5: 526–533. doi:10.1016/S2542-5196(21)00172.

We chose to use the Acton et al. (2019) study in a sensitivity analysis (scenario 3) and associated with reformulation (scenario 4). This meta-analysis was developed with three studies (Acton R, Jones A, Kirkpatrick S, Roberto C, Hammond D. Taxes and front-of-package labels improve the healthiness of beverage and snack purchases: a randomized experimental marketplace. Int J Behav Nutr Phys Act. 2019; 16:46. https://doi.org/10.1186/s12966-019-0799-0 PMID: 31113448; Acton R, Hammond D. The impact of price and nutrition labelling on sugary drink purchases: results from an experimental marketplace study. Appetite. 2018; 121:129–37. <https://doi.org/10.1016/j.appet>. 2017.11.089 PMID: 29146460; Grummon AH, Hall MG, Taillie LS, Brewer NT. How should sugar-sweetened beverage health warnings be designed? A randomized experiment. Prev Med. 2019; 121:158–66. <https://doi.org/10.1016/j>. ypmed.2019.02.010 PMID: 30772370). The study conducted by Acton et al. (2019) had the highest weight in the meta-analysis, greater than 70%, as shown in the figure below.

This figure can be found in the supplementary material of the meta-analysis (<https://doi.org/10.1371/journal.pmed.1003120>). We used a subsample of Acton et al. (2019) study with the same age group as our study. Therefore, we chose to keep the Acton et al. (2019) study in our modeling scenarios and not use the meta-analysis result due to age group adjustment.

Results

Once again, generally well presented with a few queries:

We appreciate the comment.

Table 2 and the associated text line 298-304. The text states that the mean BMI of analyzed individuals was 26.5kg/m2, which I assumed was their BMI at the beginning of the five-year modeling exercise. In the Table, it states that the BMI in base scenario is 26.5. This confused me because I thought the mean BMI of 26.5 referred to the mean BMI before any scenarios were applied. Thus I am expecting that there should be a figure that estimates the change in BMI under the base scenario where change in consumption is reduced by -20.

We thank you for your observation and added 2019 to Table 2 in the first and second lines. The remaining rows in Table 2 are the estimates after five years of FoPNL implementation.

We used the base scenario to calculate the others: we simulated scenarios 1 and 2 and made the difference with the base scenario. After that, we simulated the prevalence of obesity (time trend) and used the previous result to estimate the reduction in cases of obesity. The base scenario is part of the calculation.

In other worse, the base scenario represents a reduction in consumption of -20 after 5 years (Table 1) thus there should also be some reduction in BMI from this just as there is for scenario 1 where the expected change after 5 years is. Although the consumption of sugary beverages in Brazil is decreasing, the prevalence of obesity is increasing, and these factors were considered in all the scenarios. For this reason, we thought it made no sense to present the result of the base scenario in isolation regarding this reduction of 20kcal/per person/day.

Line 306- Figures 1: C1, C2 and C3 do not show a reduction in obesity prevalence and so appears to contradict the text. I believe the authors are referring to the reduction in scenario 1 relative to the base scenario. That is not stated in text and the particular graphs being referred to is not clear.

The modeled impact of FoPNL implementation refers to a slowdown in the increase in the prevalence of obesity. It would not be capable of reversing the problem in the country alone. This study was limited to simulating the impact of a single category of beverages on the obesity outcome in the country. We complete this excerpt with the sentence below (lines 384-386): “However, the reduction of simulated cases is unable to incline the curve of the increase in the prevalence of obesity, but it has the potential to slow down the growth.”

Line 308 refers to Figure 1 but there are six graphs attached to Figure 1. Is the 0.25 to be read from Graph A, B-1 B-2 or the series of C graphs?

We agree and appreciate the suggestion. In lines 378 and 380, we included (Fig 1, B-1) and (Fig 1, D-1), respectively.

Line 308- Can you clarify that ‘pp’ refers to percentage points? It does not appear in the graphs only in text.

Yes, it refers to percentage points. We changed all the ‘pp’ for percentage points throughout the text.

The reference in line 310 to numbers of cases of obesity avoided after FOPNL is more intuitive and informative than the percentage points more commonly referred to by authors. I recommend more use of this. Perhaps use this in the abstract to make it easier for policy makers to understand and provide more concrete thinking in terms of the impact of the FOPNL.

We included this information really to give a clear idea of the number of people who would benefit from the implementation of FoPNL. We appreciate the comment.

Discussion

This was generally very long with information that reached outside the scope of the paper insufficient focus on discussing the limitation such as the potential limitation arising from line 193.

We appreciate this observation and we included the following sentence to lines 535-538: “Furthermore, another limitation refers to the use of soda as the only sugary beverage in this study. Other sugary beverage may contain fats in their composition, which were not considered in this analysis, which could affect the results obtained in the modeling.”.

For example, the two pages spent explaining Chile’s journey through all obesity prevention policies could be summarized into one paragraph which focuses on the impact FOPNL and the multiple policies has had on obesity/overweight prevalence since that is the focus of this paper.

We thank you for this comment and agree with it. This was included in the re-discussion of the entire article in the first review.

7. PLOS authors have the option to publish the peer review history of their article (what does this mean?). If published, this will include your full peer review and any attached files.

If you choose “no”, your identity will remain anonymous but your review may still be made public.

Do you want your identity to be public for this peer review? For information about this choice, including consent withdrawal, please see our Privacy Policy.

Reviewer #2: No

Reviewer #3: No
